# Continuous‐Flow Synthesis of ZIF‐8 Biocomposites with Tunable Particle Size

**DOI:** 10.1002/anie.202000678

**Published:** 2020-03-17

**Authors:** Francesco Carraro, Jason D. Williams, Mercedes Linares‐Moreau, Chiara Parise, Weibin Liang, Heinz Amenitsch, Christian Doonan, C. Oliver Kappe, Paolo Falcaro

**Affiliations:** ^1^ Institute of Physical and Theoretical Chemistry Graz University of Technology Stremayrgasse 9 8010 Graz Austria; ^2^ Center for Continuous Flow Synthesis and Processing (CCFLOW) Research Center Pharmaceutical Engineering GmbH (RCPE) Inffeldgasse 13 8010 Graz Austria; ^3^ Institute of Chemistry University of Graz, NAWI Graz Heinrichstrasse 28 8010 Graz Austria; ^4^ Department of Chemistry and Centre for Advanced Nanomaterials The University of Adelaide Adelaide 5005 Australia; ^5^ Institute of Inorganic Chemistry Graz University of Technology Stremayrgasse 9 8010 Graz Austria; ^6^ Dipartimento di Chimica Industriale “Toso Montanari” Universita' di Bologna Viale del Risorgimento 4 Bologna Italy

**Keywords:** flow chemistry, in situ SAXS, metal–organic frameworks, MOF biocomposites, particle size

## Abstract

Zeolitic imidazolate framework (ZIF) biocomposites show the capacity to protect and deliver biotherapeutics. To date, the progress in this research area is based on laboratory batch methods. Now, the first continuous flow synthetic method is presented for the encapsulation of a model protein (bovine serum albumin, BSA) and a clinical therapeutic (α1‐antitrypsin, AAT) in ZIF‐8. The in situ kinetics of nucleation, growth, and crystallization of BSA@ZIF‐8 were studied by small‐angle X‐ray scattering. By controlling the injection time of ethanol, the particle growth could be quenched by ethanol‐induced crystallization from amorphous particles to ZIF‐8 crystals. The particle size of the biocomposite was tuned in the 40–100 nm range by varying residence time prior to introduction of ethanol. As a proof‐of‐concept, this procedure was used for the encapsulation of AAT in ZIF‐8. Upon release of the biotherapeutic from the composite, the trypsin inhibitor function of AAT was preserved.

## Graphical abstract

**The particle size** of ZIF‐8 biocomposites can be precisely tuned by controlling the injection of ethanol in the ZIF precursor solution. Owing to the abrupt amorphous‐to‐crystalline transition, an ethanol quenching method was used to adjust the particle dimension in the 40–100 nm range. This in‐flow synthesis will facilitate the application of biotherapeutic@ZIF‐8 for biomedicine.

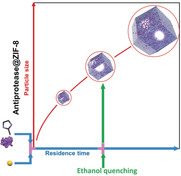

Metal–organic frameworks (MOFs) are a class of extended materials that are composed of inorganic nodes coordinated by multi‐topic organic ligands.^1^ Recently, different MOF‐based biocomposites have been studied for applications in drug‐delivery, bio‐banking and biocatalysis.^2^, ^3^, ^4^ Among the different MOFs explored for biotechnology and biomedicine, zeolitic imidazolate framework 8 (ZIF‐8) has been extensively studied as its components can self‐assemble around bioentities, under biocompatible conditions, to form a protective crystalline coating.^5^, ^6^, ^7^ Typically, ZIF‐8 biocomposites are synthesized by mixing selected biomacromolecules and the ZIF precursors (2‐methylimidazole, HmIM, and Zn^2+^) in water.^3^ By varying the concentration and molar ratios of ZIF precursors and protein, a number of different structure phases can be prepared (for example, ***sod***, ***dia***, ***kat, ZIF‐C***).^8^, ^9^ The most extensively investigated topology is sodalite (***sod***)^10^ as it has been shown to afford crystalline microporous materials that can protect encapsulated proteins from inhospitable environments.^5^, ^6^, ^7^, ^11^, ^12^ Furthermore, the ***sod*** ZIF‐8 coating can be degraded under mild conditions (mild acidic conditions, phosphate ions or chelating agents) allowing for triggered release of the encapsulated biomolecule with retention of its native activity.^13^, ^14^

To advance research of ZIF‐8 biocomposites for application to biomedicine, such as drug delivery, there is a need for the larger scale production of particles with controlled size.^15^, ^16^, ^17^ For example, a number of studies have highlighted how the dimensions of nanoparticle‐based systems can influence blood‐circulation time, cellular uptake and mechanism of internalization.^15^

In recent years, flow chemistry has become established as an effective technology for the scalable synthesis of fine chemicals, active pharmaceutical ingredients, and functional materials.^18^, ^19^ Enhanced and scale‐independent heat transfer and mass transfer allow straightforward scalability by, for example, lengthening the processing time. Another key advantage of flow processing is that precise and reproducible access to well‐defined (typically very short) reaction times can be achieved by varying the respective reactor volumes or flow rates.^20^, ^21^ Recently, it has been demonstrated that MOFs can be prepared with controlled particle size using flow technology.^22^, ^23^, ^24^, ^25^, ^26^, ^27^, ^28^ Specifically, ZIF‐8 has been produced in flow using a variety of equipment and procedures.^29^, ^30^, ^31^, ^32^ However, the concept of tuning particle size by controlling residence time prior to ethanol‐induced crystallization has not been reported for any MOF. Furthermore, the preparation of biomacromolecules@ZIF‐8 in flow has not been described before.

Herein, we show that flow synthesis can be used as a scalable method to prepare BSA@ZIF‐8 with controlled particle size in the 40 to 100 nm range, by modifying the residence time prior quenching with ethanol. This procedure was applied to the synthesis of a ZIF‐8‐based composite of a clinical biotherapeutic (protease inhibitor α1‐antitrypsin, AAT) with a size suitable for intravenous drug delivery applications.^15^ Further, we released AAT from the ZIF‐8 host and showed that bioactivity was preserved.

To develop a continuous flow procedure and to understand the growth kinetics for ZIF‐8 based composites we used BSA as model protein (Supporting Information, Section S2.1, 4).^5^, ^8^, ^33^, ^34^ The nucleation and growth of BSA@ZIF‐8 was examined by a synchrotron time‐resolved SAXS study using a stopped‐flow set‐up (Supporting Information, Sections S2, S3). Injection of the Zn(OAc)_2_ and BSA‐HmIM solutions triggered the acquisition system of rapid SAXS data collection with a time resolution of 100 ms. The quantity *Q̃* (Supporting Information, Section S2.1), related to the invariant of the scattering curve and sensitive to changes in particles volume fraction and electron density contrast, increased during the first 3 min. This is attributed to rapid growth of amorphous particles with dimension larger than the set‐up resolution limit (>35 nm, *q*_min_=0.1 nm^−1^ thus no Guinier regime and no diffraction peaks were detected; Figure 1; Supporting Information, Figure S7).^35^ The amorphous particles are composed by BSA and by HmIM coordinated to Zn^2+^, as determined via FTIR^36^ (Supporting Information, Figure S9). After 3 min, the (110) diffraction peak of ZIF‐8 was observed, and its increase in intensity was accompanied by a decrease of *Q̃* (Figure 1 a). This trend can be explained by the conversion of amorphous particles (high density) into ZIF‐8 crystals (low density),^37^, ^38^ resulting in the overall reduction of *Q̃*.^39^ Bustamante et al. demonstrated that alcoholic solvents facilitate the crystallization of ZIF‐8.^40^ We hypothesized that the rapid injection of ethanol could quench the growth of amorphous particles and engender crystallization of ZIF‐8. To examine the role of ethanol in ZIF‐8 crystallization, in the stopped‐flow set‐up we mixed aqueous solutions of BSA/HmIM and Zn^2+^ and then, after 19 ms, we added an equal volume of ethanol.

**Figure 1:**
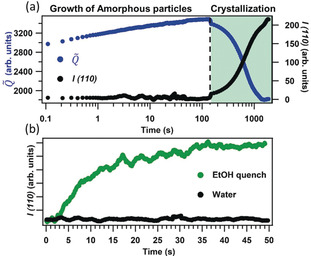
a) Time evolution of the integrated intensity of the (110) reflection of ZIF‐8 (*I(110)*) and of *Q̃* (0.1–0.7 nm^−1^ range) calculated from time‐resolved SAXS synthesis of BSA@ZIF‐8 in water. b) Time evolution of the integrated intensity of the (110) diffraction peak of *sod* ZIF‐8 (*I(110)*) during the first 50 s of the synthesis of BSA@ZIF‐8 in water (black) and the synthesis quenched with ethanol after 19 ms (green).

After 2 s from the injection of ethanol, the (110) diffraction peak of ***sod***‐ZIF‐8 was detected (Figure 1 b; Supporting Information, Figure S8). The integrated intensity of this peak over time is plotted in Figure 1 b, and shows that a plateau is reached after 35 s. We note that the observation of the 110 reflection was 90 times faster than neat water. The average crystallite size obtained from the ethanol quench was 17 nm while the non‐quenched synthesis yields crystallites larger than 200 nm. These data suggest that ethanol both triggers the crystallization of BSA@ZIF‐8 biocomposites and quenches the particle growth.

Further, we used a simple microfluidic setup composed of Y‐ and T‐mixers (Figure 2 a; Supporting Information, Sections S2.2, S5) to ensure that replication of biocomposite synthesis could be achieved without the need for specialized equipment. This continuous flow set‐up was initially used to study the influence of the ethanol/water flow rate ratio on the crystal size, we fixed the residence time to 0.33 s and varied the flow rate of ethanol (Supporting Information Section 5.5). The trend is reported in Figure 2 b (Supporting Information, Table S2) and shows that a higher ethanol flow rate results in smaller ***sod*** crystallites, with a minimum size of ca. 50 nm (Figure 2 b; Supporting Information, Table S2). We examined the crystal sizes by scanning electron microscopy (SEM; Supporting Information, Figures S18, S19), and for the highest ethanol/water ratio (that is, 1.6) we observed a broader particle size distribution. Accordingly, we decided to use a 1:1 flow rate ratio for a better control over the particle size distribution. Other quenching methods (for example, crystal modulators,^41^ 1‐methyl imidazole,^42^ Supporting Information, Sections 5.2, 5.3) were also examined, but were found to be comparatively ineffective.

**Figure 2:**
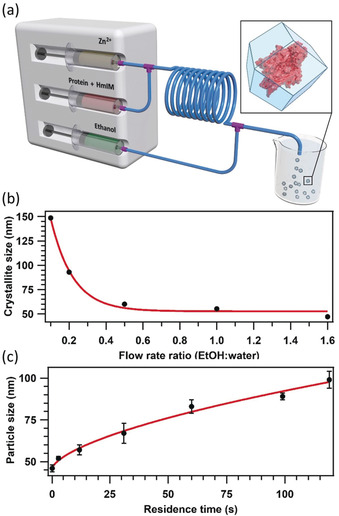
a) View of the microfluidic setup, where the residence time prior quenching can be varied by changing the length of the reactor or flow rates. b) Average crystallite size of BSA@ZIF‐8 obtained, versus the ethanol flow rate employed. The red line is the fitted exponential decay (crystallite size=*a*+b*e ^−*x*/*t*^, with *a*=53±3, *b*=220±30, *τ*=0.6±0.1, *x*=flow rate ratio, *R* ^2^=0.98). c) Average particle size obtained from AFM topography as a function of the residence time, including a power law fit of the experimental data (particle size=*a*+b*x ^*c*^, with *a*=45±3, *b*=3±1, *c*=0.6±0.1, *x*=residence time, *R* ^2^=0.97).

Next, we examined the influence of the residence time when using a 1:1 ethanol/water ratio. The prepared biocomposites were studied by atomic force microscopy (AFM) and SEM (Figure 2 c; Supporting Information, Section S6). By varying the residence time from 0.33 to 120 s we could synthesize BSA@ZIF‐8 crystals with particle sizes ranging from 40 to 100 nm. The AFM data were fitted with a power law that can predict the required residence time to obtain particles of a desired size (Figure 2 c; Supporting Information, Section 6). For selected samples (residence times=0.33 s, 30 s and 120 s for samples B1, B2 and B3, respectively), we further investigated the structural and physicochemical properties of the biocomposites. X‐ray diffraction (XRD) confirmed the ***sod*** topology of ZIF‐8 for all measured samples (Figure 3 a; Supporting Information, Figure S23). Moreover, the crystallite size calculated from (110) diffraction peak of ZIF‐8 (37, 59, and 95 nm for samples B1, B2, and B3, respectively; Supporting Information, Table S3) are in agreement with the particle size calculated from their AFM micrographs, suggesting the formation of single crystal particles of BSA@ZIF‐8. Additionally, N_2_ sorption isotherms at 77 K confirmed the presence of permanent microporosity and a dependence of the gate opening pressure on the particle size (Supporting Information, Figure S24).^30^, ^43^ After washing with water, ethanol, and SDS^8^ (Supporting Information, Section 4.1), the encapsulation of BSA was confirmed via Fourier transform infrared (FTIR) spectroscopy, which show the characteristic BSA bands (for example, amide I at 1700‐1610 cm^−1^ and amide II at 1595‐1480 cm^−1^; Figure 3 b; Supporting Information, Figure S23) in addition to the typical fingerprint of ***sod*** ZIF‐8.^44^, ^45^ Finally, the loading of BSA for samples B1, B2 and B3 was determined to be 5.1±0.6, 5.2±0.6 and 5.8±0.4 wt %, respectively, by ICP‐MS (Supporting Information, Table S4).

**Figure 3:**
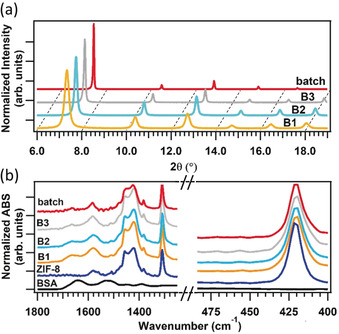
XRD patterns a) of BSA@ZIF‐8 synthesized in batch and in flow (B1, B2, B3). FTIR spectra b) of BSA@ZIF‐8 synthesized in batch and in flow (B1, B2, B3) and of ZIF‐8 and BSA.

To demonstrate the scalability of the method, we synthesized BSA@ZIF‐8 with average particle size of 60 nm in continuous flow for 5 h (Supporting Information Section 8). During this time the calculated standard deviation of the average particle size was only 3 nm; the measured productivity was 2.1 g h^−1^, a value that is comparable with the flow preparation of pure MOFs on lab scale.^26^ Next, we studied the long‐term stability of BSA@ZIF‐8 in the stock solution (as collected from the flow set‐up) by AFM. In this case we selected the smaller particles as they are typically less stable than larger particles;^46^, ^47^ our analysis demonstrated that the particle size distribution remained unchanged over a two‐week period (Supporting Information, Section S6.1).

We applied the procedure developed for BSA@ZIF‐8 to synthesize biocomposites of α_1_‐antitrypsin, AAT. AAT is a member of the serine protease inhibitor (serpin) superfamily and is currently under investigation as a biotherapeutic for the treatment of several neutrophilic diseases, for the control of inflammatory, immunological, and tissue‐protective responses.^48^, ^49^, ^50^, ^51^, ^52^ Three different samples were synthesized using the flow setup, employing the residence times used for the preparation of BSA@ZIF‐8 biocomposites (Supporting Information, Section S7). The crystallite sizes calculated from the (110) diffraction peak for the different samples (Supporting Information, Figure S27; 40, 59, and 87 nm for samples A1, A2, and A3, respectively) are analogous to those calculated for the BSA@ZIF‐8 samples. However, the particle sizes measured by AFM (Supporting Information, Figure S26; A1=93 nm, A2=109 nm, A3=179 nm) are larger than the particle size observed for BSA (B1=46 nm, B2=67 nm, B3=99 nm). This could be explained by the formation of crystalline clusters suggesting that that the type of proteins influences the structure of the final biocomposite.^5^ The encapsulation of AAT was confirmed by the presence of amide I and amide II bands (Supporting Information, Figure S27) via FTIR spectroscopy.^44^ The loading of AAT for samples A1, A2, and A3 was determined to be 4.2±0.3, 4.1±0.7, and 3.1±0.6 wt % by ICP‐MS (Supporting Information, Table S6).

To ascertain whether the flow chemistry procedure (that is, ethanol quench)^53^ and the ZIF‐8 chemical environment^54^ affect the AAT protein structure (that is, denaturation) we studied the activity of the released bio therapeutic.^55^ Thus, the ZIF matrix of AAT@ZIF‐8 was dissolved in 1 mm HCl and the released AAT exposed to a trypsin solution and incubated at 4 °C for 30 min. Then, the protease activity of trypsin was evaluated using a standard colorimetric assay (Supporting Information, Section S2.1). Importantly, the resulting data confirmed that the released AAT was successfully inhibiting protease, and, thus remains active after release from the ZIF matrix (Figure 4).^55^ Additionally, when AAT@ZIF‐8 crystals were exposed to trypsin at pH 7.0, the proteolytic activity was fully retained (Supporting Information, Figure S29). These results confirmed that AAT is encapsulated within the ZIF‐8 particles and the inhibition of trypsin occurs only when AAT is released from the MOF biocomposite. In general, as ethanol can denature some proteins,^53^, ^56^ the compatibility of each specific biomacromolecule with the quenching agent should be examined.

**Figure 4:**
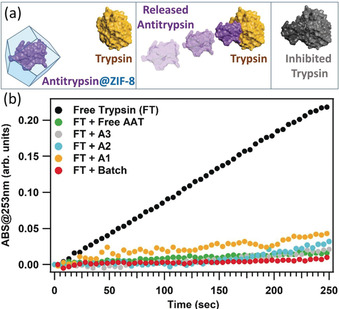
a) View of free trypsin and of the encapsulated AAT (left), of the AAT released after the ZIF‐8 dissolution (middle), and of the trypsin inhibited by the interaction with AAT. b) Trypsin protease activity results of trypsin and of trypsin exposed to AAT and to AAT released from AAT@ZIF‐8 samples (batch and flow syntheses).

In conclusion, we report the first synthesis of ZIF‐8‐based biocomposites with sodalite topology in continuous flow. By using a simple experimental setup, the particle size can be precisely tuned by controlling the residence time prior to injection of ethanol to the mixed ZIF precursor solution. Owing to the abrupt amorphous‐to‐crystalline transition, an ethanol quenching method was used to adjust the particle dimensions in the 40–100 nm range. The general study was performed using BSA as model protein and we demonstrated that 60 nm BSA@ZIF‐8 particles could be continuously prepared for 5 h. Then we applied the flow system to test for the encapsulation of AAT, a biotherapeutic with anti‐inflammatory and immunomodulatory properties. AAT@ZIF‐8 samples with 90, 110 and 180 nm were prepared and after dissolution of the ZIF matrix, the protease inhibitor fully retained its bioactivity. The continuous flow synthesis of ZIF‐8‐based composites afforded a control over the particle size that is suitable for intravenous drug delivery administration (particle size ≤200 nm). We believe that this synthetic method will facilitate the application of biotherapeutic@ZIF‐8 for biomedicine. It is anticipated that the precise control over the particle dimension and topology could also expedite the use of enzyme@ZIF‐8 for biocatalytic applications.

## Conflict of interest

The authors declare no conflict of interest.

## Supporting information

As a service to our authors and readers, this journal provides supporting information supplied by the authors. Such materials are peer reviewed and may be re‐organized for online delivery, but are not copy‐edited or typeset. Technical support issues arising from supporting information (other than missing files) should be addressed to the authors.

Supplementary
