# Supplementary material for: Continuous‐Flow Synthesis of ZIF‐8 Biocomposites with Tunable Particle Size
Source: Angew Chem Int Ed Engl. 2020 Mar 17;59(21):8123–7. doi: 10.1002/anie.202000678 (PMC7318291; doi:10.1002/anie.202000678)
Supplement: Supplementary file 1 — Supplementary [file ANIE-59-8123-s001.pdf]

Angewandte Chemie
Eine Zeitschrift der Gesellschaft Deutscher Chemiker

Supporting Information

Continuous-Flow Synthesis of ZIF-8 Biocomposites with Tunable Particle Size

Francesco Carraro, Jason D. Williams, Mercedes Linares-Moreau, Chiara Parise, Weibin Liang, Heinz Amenitsch, Christian Doonan, C. Oliver Kappe, and Paolo Falcaro**

anie_202000678_sm_miscellaneous_information.pdf

1. Contents

1. Contents 1
2. General Information 2
   2.1. Materials and Methods 2
   2.2. Flow Reactor Setup 5
   2.3. Mixing Time Characterization 6
3. Time-resolved SAXS results 9
4. General methods 12
   4.1. BSA@ZIF-8 synthesis in flow 12
   4.2. AAT@ZIF-8 synthesis in flow 12
5. Quenching system optimization experiments 14
   5.1. BSA@ZIF-8 synthesis in flow without quench 14
   5.2. Dilution with water 14
   5.3. Quench with 1-methylimidazole 15
   5.4. Quench with ethanol 16
   5.5. Optimization of ethanol flow rate 18
6. Analysis of BSA@ZIF-8 produced using varied residence times 19
   6.1. Measure of BSA@ZIF-8 stability in stock solution 23
7. Analysis of AAT@ZIF-8 produced using varied residence times 25
8. Scalability of the synthetic method 28
9. References 32

2. General Information

2.1. Materials and Methods

BSA was purchased from Sigma Aldrich, lyophilized powder, >96%, product code: 1002695029. 2-Methylimidazole (HmIM) was purchased from Sigma Aldrich, 99% purity, product code: M50850. $Zn(OAc)_2 \cdot 2H_2O$ was purchased from Sigma Aldrich, product code: 1.08802.0250. Ethanol was purchased from Merck, EMSURE®, for analysis, denatured with ~1% methyl ethyl ketone, product code: 1.00974.2500. 1-Methylimidazole (1-mIM) was purchased from Sigma Aldrich, product code: 67560. *N*α-Benzoyl-L-arginine ethyl ester hydrochloride (BAEE) was purchased from TCI, >98%, product code: B0853. α1-Antitrypsin from human plasma was purchased from Sigma Aldrich, lyophilized powder, product code: 1002697009. Trypsin from porcine pancreas was purchased from Sigma Aldrich, lyophilized powder, 1000-2000 BAEE units/mg, product code: 1002666851.

SEM micrographs were collected using Tescan VEGA 3 SEM with tungsten source filament working at 20 kV. Prior the analysis the powder samples were drop cast on a piece of Si (100) and sputter-coated with Gold.

Atomic Force Microscopy measurements were performed using an Anton Paar Tosca 400 AFM. A 1:1000 dilution of the stock solution in ethanol was made and drop cast on clean Si (100) substrates. Samples were washed with ethanol and dried to eliminate residual HmIM and unencapsulated biomacromolecule from the ZIF-8 particles. AFM topography and phase contrast images were acquired in tapping mode with MikroMasch HQ:NSC15/AL BS silicon probes (resonance frequency 325 kHz).

FT-IR spectra were recorded on a Bruker ALPHA spectrometer using the ATR accessory with a diamond window in the range 400 – 4000 cm^{-1}.

XRD patterns were acquired using a Rigaku SmartLab II equipped with a Cu anode (λ=1.5406 Å) and operating at 9 kW. The analysis of the crystallite size of ZIF-8 were performed using the Powder XRD plugin (Scherrer method; LaB_6 was used to calculate the instrumental broadening factor) of the SmartLab Studio II software (Rigaku).

Time-resolved Small Angle X-Ray Scattering (SAXS): Time-resolved SAXS data were collected on the SAXS beamline at the ELETTRA synchrotron light source.[1,2] We operated at photon energy of 8 keV covering the range of momentum transfer, $q = 4\pi \sin(\theta)/\lambda$, between 0.1 and 6.8 nm^{-1}. We monitored the kinetic of ZIF-8 and BSA@ZIF-8 nucleation and growth using a commercial stopped flow set-up (Bio-Logic, Grenoble, France, Figure S3) especially designed for Synchrotron Radiation SAXS investigations and a Pilatus3 1M, Dectris Ltd detector (Baden, Switzerland; sample to detector distance: 1260 mm, as determined with a

silver behenate calibration sample). The quantity $\tilde{Q}$ is related to the invariant of the scattering curve, but it is calculated only over an interval of the measurement regime and is defined as $\tilde{Q} = \int_{q_1}^{q_2} dq * q^2 * I(q)$, where $q_1 = 0.1\, nm^{-1}$ and $q_2 = 0.7\, nm^{-1}$. As a consequence, $\tilde{Q}$ represents a measure of structural changes occurring in the system, which must be discussed taking into account the selected q-window. In this case, a decrease of $\tilde{Q}$ can be associated to a decrease of the density (**Figure 1a**). This trend can be explained by considering that the non-porous amorphous ZIF-8 is denser than the porous sod ZIF-8.[3] The increase of the pores within this material can be attributed to the reduction of the electron density. This explains the decrease of $\tilde{Q}$ reported in **Figure 1a**. The integrated scattering intensity $I(110)$ is defined as $I(110) = \int_{q_1}^{q_2} dq * I(q)$, where $q_1 = 4.5\, nm^{-1}$ and $q_2 = 6\, nm^{-1}$. The analysis of the crystallite size of ZIF-8 synthesized with the stopped flow set-up were performed using the Powder XRD plugin (Scherrer method; silver behenate was measured at the SAXS beamline and was used to calculate the instrumental broadening factor) of the SmartLab Studio II software (Rigaku).

Protease Inhibitor activity test

To test the protease inhibitor activity of α1-Antitrypsin, we exposed α1-Antitrypsin (stock solution prepared from the lyophilized powders or released from AAT@ZIF-8 biocomposites) to a solution containing Trypsin. Then, we evaluate the enzymatic activity of Trypsin following the enzymatic colorimetric assay of Trypsin (EC 3.4.21.4)[4] and using BAEE as substrate. Briefly, for each test, 0.333 mL of the AAT@ZIF-8 stock solution (as collected from the flow setup) was washed three times with a water-ethanol mixture (1 to 1 volume ratio) and then twice with water. The washed powders were dispersed in 0.495 mL of 1 mM HCl aqueous solution at 4 °C for 1 h to dissolve the MOF (800 rpm, thermomixer; as control we tested a sample of pure ZIF-8, **Figure S30**). Considering the total encapsulation of AAT, the protein concentration of this solution is 0.111 mg/mL. Then, 0.113 mL of this solution was mixed with 0.075 mL of a 1 mM HCl cold water solution containing 0.0075 mg of trypsin (prepared fresh) and the mixture was aged on a thermomixer (800 rpm) for 30 min at 4 °C prior the enzymatic tests. For the test of the pure AAT, a 0.111 mg/mL AAT in 1 mM HCl aqueous solution was prepared, stored at 4 °C for 1 h and then mixed (0.113 mL) with the trypsin solution (0.075 mL). For the test of the pure trypsin, 0.113 mL of 1 mM HCl aqueous solution was mixed with the trypsin solution (0.075 mL). As control experiments and to prove the encapsulation of AAT, we tested the activity of trypsin exposed to the not-degraded AAT@ZIF-8 crystals and to pure ZIF-8 (**Figure S29**). To test the trypsin activity, we used the same experimental conditions reported above. The unique difference is that trypsin and MOF particles were dispersed in water at pH=7 and not in HCl: in these conditions, ZIF-8 does not degraded.[5] For the AAT@ZIF-8 sample we hypothesized ATT should not be able to interact with trypsin. For all

samples, after 30 mins, the solid was separated by the supernatant by centrifugation (5 min, 21000 rcf). The supernatants were tested with the trypsin assays and the activity of trypsin was fully retained (**Figure S29**). These results confirmed that AAT is encapsulated within the ZIF-8 particles. Furthermore, pure ZIF-8 does not influence the trypsin activity.

Inductively Coupled Plasma Mass Spectrometer (ICP-MS)

ICP-MS was performed on an Agilent 8900x QQQ-ICP-MS. The free proteins or protein@ZIF-8 composites (approximately 1 mg) were dispersed in a solution of HNO_3/HCl (0.25 mL of 70% HNO_3 (Ajax) and 0.1 mL of 37% HCl (Chem Supply)) and stored in Eppendorf tubes at room temperature overnight. The mixture was then centrifuged to remove any particulates in the supernatant. Thereafter, the clear supernatant (0.4 mL) was diluted to a final volume of 5 mL with H_2O for ICP-MS analysis. The amount of protein within the sample was calculated according to a standard calibration curve for sulfur (prepared from sulfur standard solution from Sigma-Aldrich, Sulfur Standard for ICP (TraceCERT, 1000 mg/L S in H_2O)) and to the quantification of sulfur in the free proteins.

2.2. Flow Reactor Setup

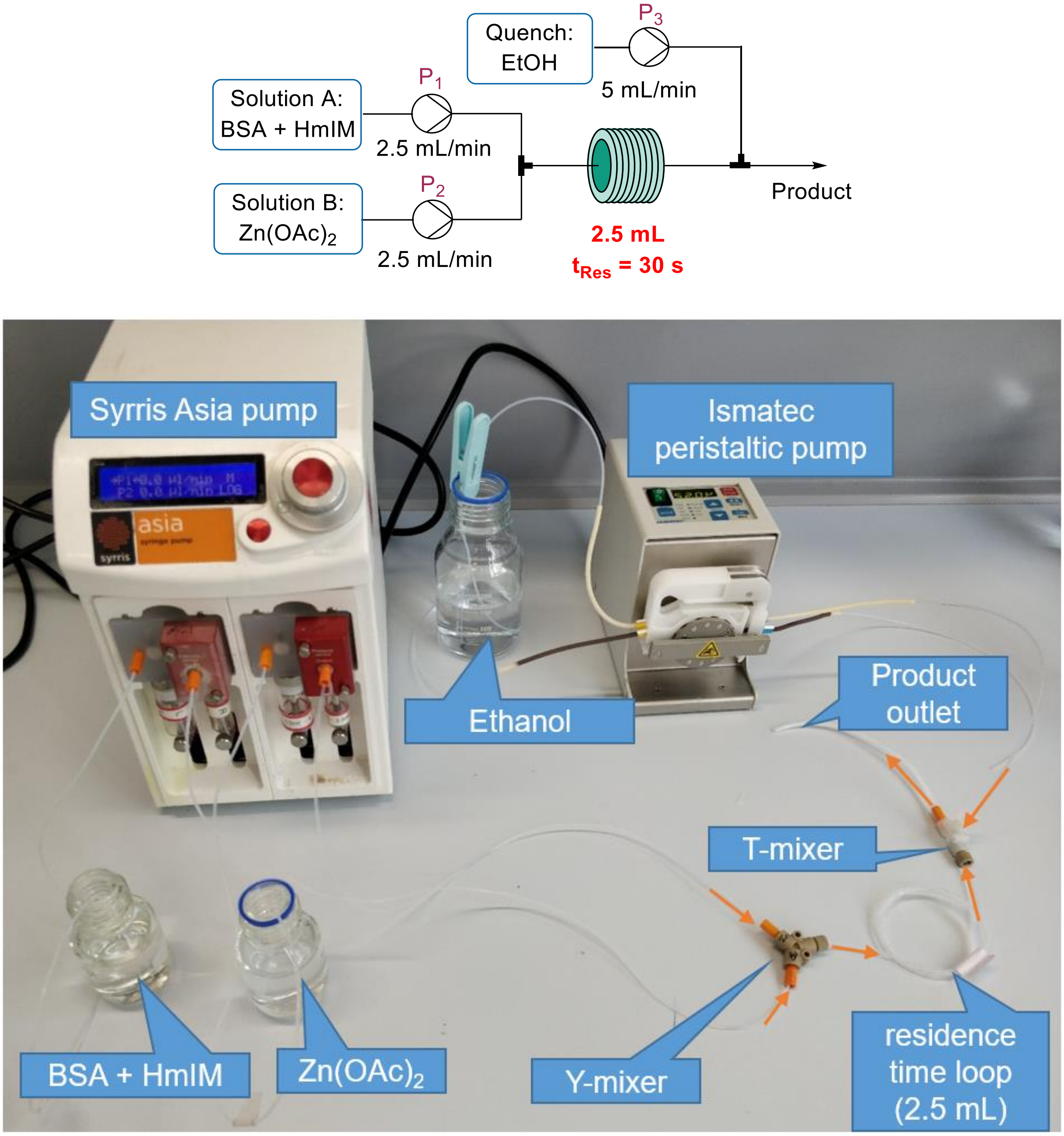

Figure S1. Top: Schematic view of setup used for MOF synthesis in flow, equipped with a 2.5 mL residence time coil. **Bottom:** Photograph of the setup depicted in the schematic.

Pumps: A Syrris Asia reciprocating syringe pump was used to deliver both ZIF-8 component streams (BSA + HmIM and $Zn(OAc)_2 \cdot H_2O$). The pump was equipped with "red" syringes (2.5 mL / 5 mL volume; 0.05-10 mL/min flow rate; wetted parts: PTFE and glass).

An Ismatec peristaltic pump (MS-2/08-160) was used to deliver the ethanol stream. The pump was equipped with a Tygon MHLL/SC0713 peristaltic tube.

General connections: Connections between the pumps and mixer were made of 1/16” outer diameter (0.8 mm inner diameter) PFA tubing, connected with 1/4” PTFE finger tight fittings.

Reactors of 0.0275–5 mL volume were made using 1/16” outer diameter PFA tubing (0.8 mm inner diameter). The reactor of 10 mL volume was made using 1/8” outer diameter PFA tubing (1.6 mm inner diameter).

Mixers: To mix the BSA/HmIM and $Zn(OAc)_2$ solutions, a Y-mixer (PEEK), with a 0.5 mm aperture, was used. To introduce the ethanol stream, a T-mixer (PTFE), with a 0.5 mm aperture, was used.

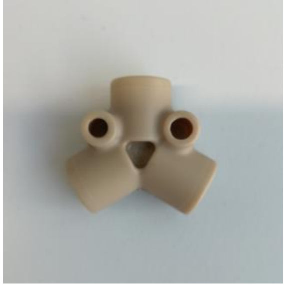

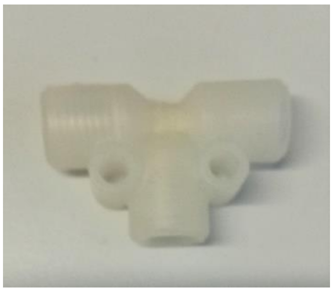

Figure S2. Images of the simple mixing connectors used in this work. **Left:** PEEK Y-mixer; **Right:** PTFE T-mixer.

Sample loop: To conserve material, experiments using antitrypsin were performed with a sample loop for injection of the antitrypsin + BSA solution. Upchurch 6-way switching valve (part # V-450, https://www.idex-hs.com/store/injection-valve-2-postion-6-port-040-black.html), with a 2 mL sample loop made from PFA tubing installed (1/16” outer diameter, 0.8 mm inner diameter).

2.3. Mixing Time Characterization

In order to determine the time required for complete mixing in this simple system, analysis was carried out using the Villermaux-Dushman protocol. This test functions through a system of two competing reactions – an instantaneous neutralization, versus the acid-mediated generation of iodine from iodide and iodate ions. The extent at which iodine is generated can be used to determine the time required for complete mixing of the system.[6]

Experimentally, this was realized by the setup shown below (**Figure S3**). A 250 mL quantity of buffer solution and $HClO_4$ solution were made up, then pumped through reciprocating syringe pumps (Syrris Asia) at equal flow rates. After the mixer, a 2.5 mL length of tubing was added to ensure complete reaction, prior to flowing through a UV/vis cell (Ehrfeld, part #0551-2-0001-F) with an optical path length of 1 cm. UV/vis data were recorded using a fiber-coupled Avantes Starline AvaSpec-2048 spectrometer, with an Avantes AvaLight-DHc lamp as the light source. Collection parameters: 15 ms integration time, 100× averaging (1.5 s measuring time per spectrum). These spectra were processed using Avasoft 8.7 software

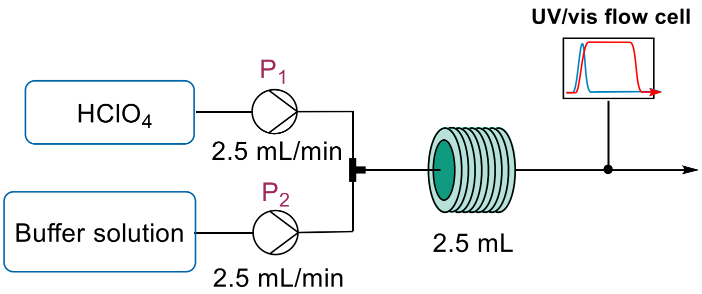

Figure S3. Schematic of the experimental setup for Villermaux-Dushman mixing characterization.

The solution concentrations were as follows, using concentrations "2c" from the referenced study[6], but using perchloric acid in place of H2SO4[7]:

Table S1. Solution concentrations for Villermaux-Dushman studies.

**$HClO_4$ solution**		**Buffer solution**	
**Component**	**Concentration (M)**	**Component**	**Concentration (M)**
$HClO_4$	0.02	KI	0.016
		KIO_3	0.003
		$B(OH)_3$	0.045
		NaOH	0.045

First, the mixing speed at the Y-mixer (mixing point for BSA/HmIM and $Zn(OAc)_2$ solutions) was tested, using flow rates in the range of those used throughout the study. The results are shown below, and a mixing time of 0.30 s was determined (**Figure S4**).

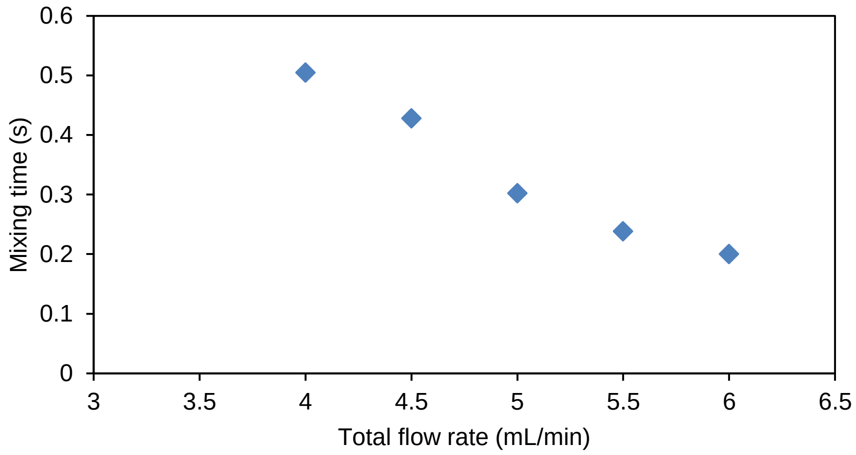

Figure S4. Results of Villermaux-Dushman analysis of Y-mixer.

The T-mixer (mixing point for MOF solution and ethanol) was also tested in the same system, using flow rates in the range of those used throughout the study. The results are shown below, and a mixing time of 0.23 s was determined (**Figure S5**).

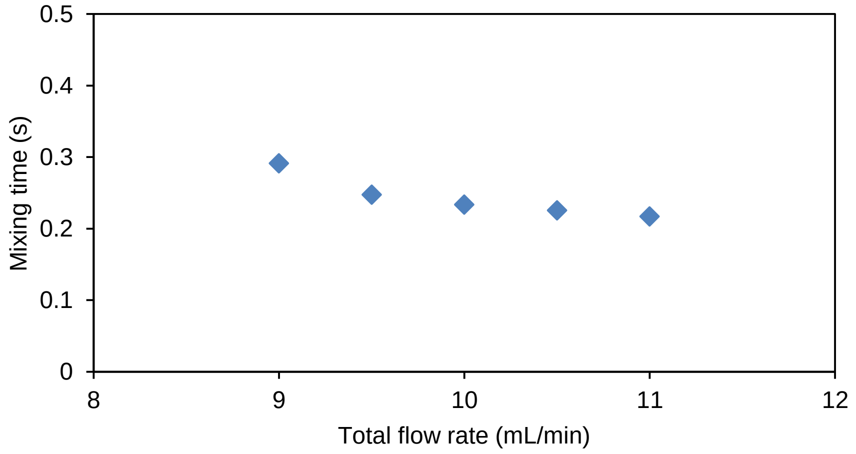

Figure S5. Results of Villermaux-Dushman analysis of T-mixer.

3. Time-resolved SAXS results

We monitored the kinetic of ZIF-8 and BSA@ZIF-8 nucleation and growth using a commercial stopped flow set-up (**Figure S6**). Two independently driven syringes were filled respectively with the $Zn(OAc)_2$ solution and the ligand (or ligand + protein) solution. Two step-motors control the volumes mixed and injected in a 1 mm quartz capillary placed in the X-Ray beam (the mixing/injection process lasts few ms). The concentration of the precursors and the volume ratio between the two solutions were set to maintain the conditions used for the syntheses in flow (see section 4).

For each experiment, a total volume of 800 μL was injected. The start of the mixing sequence is triggered from the X-ray data-acquisition system, which took images with a time resolution of 100 ms. All the experiments were performed at RT. A water solution of $Zn(OAc)_2$ was measured in order to assess and subtract the background from the data. The resulting two-dimensional images were radially integrated to obtain a 1D pattern of normalized intensity versus scattering vector q.

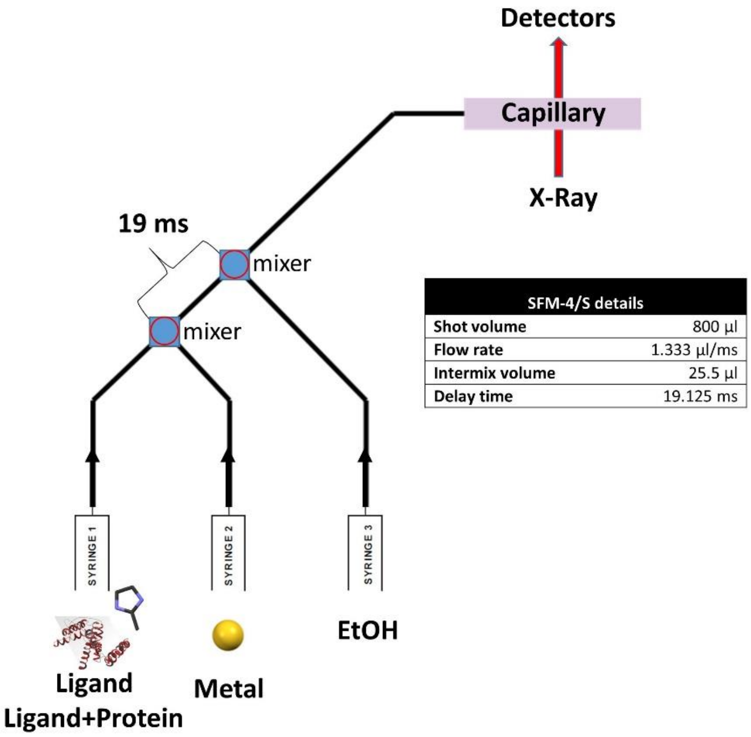

SFM-4/S details	
Shot volume	800 μl
Flow rate	1.333 μl/ms
Intermix volume	25.5 μl
Delay time	19.125 ms

Figure S6: Schematic view of the stopped-flow set-up used with *in situ* SAXS analysis.

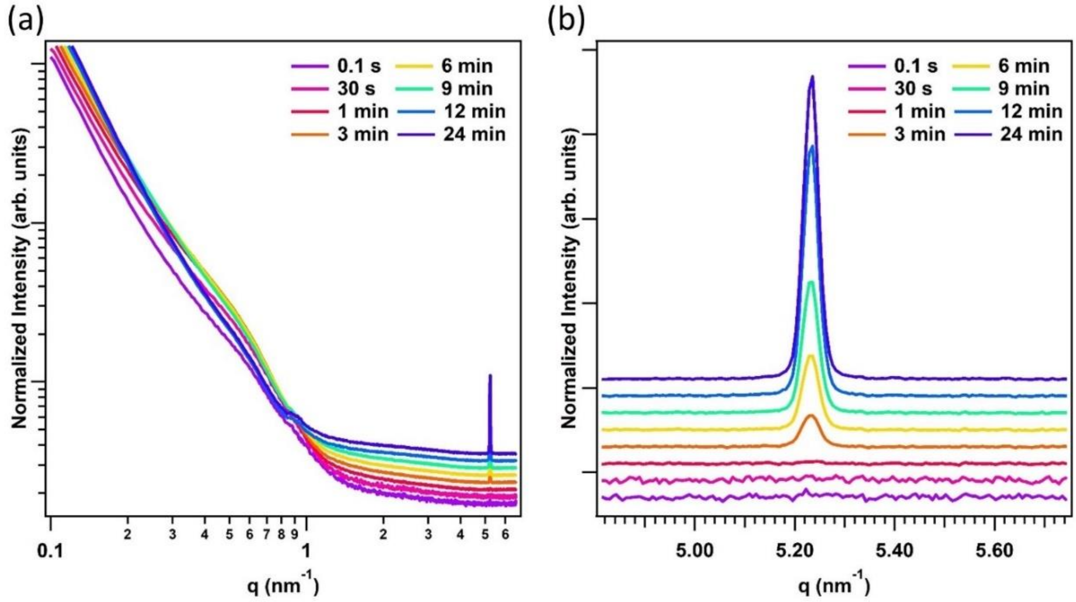

Figure S7. Time-resolved SAXS patterns (a) collected during the synthesis of BSA@ZIF-8 in water. In (b) the time evolution of the intensity of the (110) diffraction peak of ***sod*** ZIF-8 is highlighted.

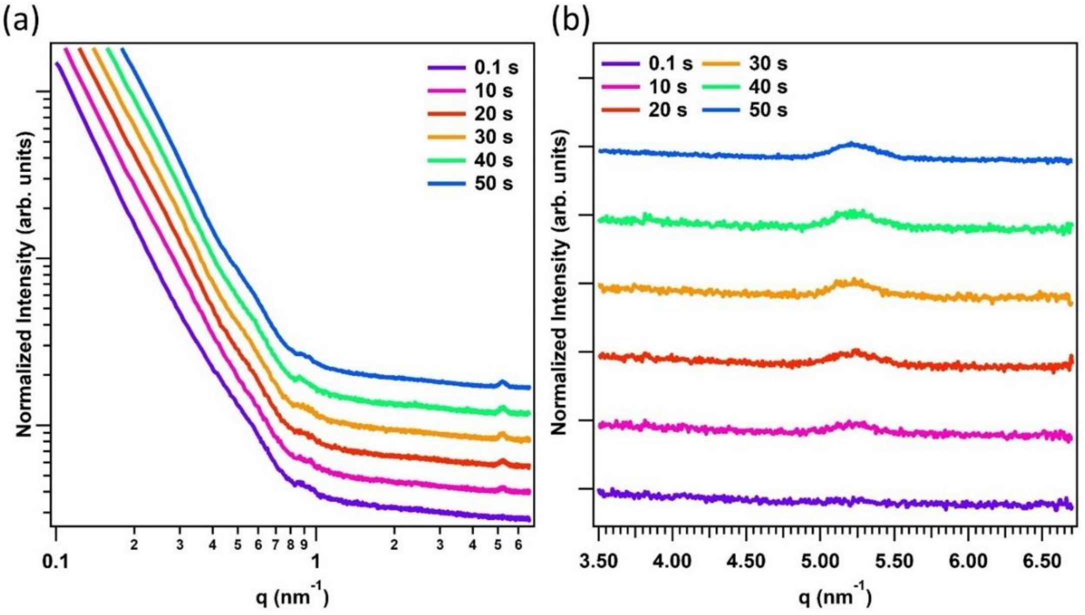

Figure S8. Time-resolved SAXS patterns (a) collected during the synthesis of BSA@ZIF-8 quenched with ethanol after 19 ms. In (b) the time evolution of the intensity of the (110) diffraction peak of ***sod*** ZIF-8 is highlighted.

We investigated the chemical nature of the amorphous particles via IR spectroscopy. The solid product was separated from the reaction mixture 30 s after the mixing of the reagents (BSA, HmIm, Zn2+) via centrifugation (20 s, 15000 rpm). We chose 30 seconds as the entire procedure (synthesis, separation and measurement) should be within the lifetime of the amorphous phase (3 minutes, see **Figure S9**). The recovered material was washed once with water (20 s, 21000 rcf) and the wet powder analyzed via FTIR spectroscopy (ATR mode – Alpha Bruker). In the collected spectra, we could observe modes that are attributed to HmIM coordinated to zinc (e.g. Zn-N stretching 420 cm^{-1}, C-N stretching 1145 cm^{-1}, ring stretching 1300-1460 cm^{-1}) and BSA (e.g. Amide I at 1700-1610 cm^{-1} and Amide II at 1595-1480 cm^{-1}). Furthermore, the FTIR spectrum of this sample is very similar to previously reported amorphous protein@ZIF-8 systems.[8]

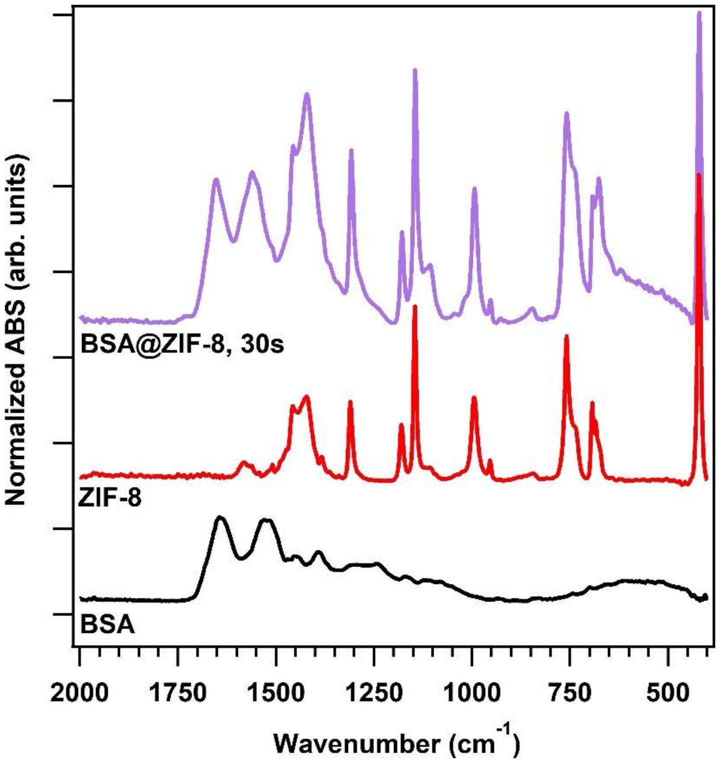

Figure S9: FTIR spectra of BSA, ZIF-8 and of a BSA@ZIF-8 sample recovered and washed 30 s after the mixing of the reagents.

4. General methods

4.1. BSA@ZIF-8 synthesis in flow

For all experiments described using BSA, a previously published recipe[9] was used:

Solution A: BSA (33 mg) and 2-methylimidazole (5.25 g, 63.9 mmol) were added to a 50 mL volumetric flask. The solution was made up using deionized water.

Solution B: $Zn(OAc)_2 \cdot 2H_2O$ (0.878 g, 4.0 mmol) was added to a 50 mL volumetric flask. The solution was made up using deionized water.

Procedure:

Solutions A and B were pumped directly through the reciprocating syringe pump at a flow rate of 2.5 mL/min each. Ethanol was pumped through the peristaltic pump at a flow rate of 5 mL/min. The reactor was allowed to equilibrate for 3 residence times, before collecting samples of 2 mL volume at the outlet.

Between each sample collection, the tubing was cleaned by injecting a 1 mL portion of acetic acid, followed by 2 portions of water.

For comparison, the synthesis in batch were performed by mixing 1 mL of solution A and 1 mL of solution B.[9] After 24h, the powder was recovered via centrifugation (5 min, 21000 rcf), washed (dispersion and centrifugation cycles) 3 times with deionized water and ethanol mixture (1 to 1 volume ratio), 1 time with 10% Sodium dodecyl sulfate (SDS) aqueous solution, 3 times with deionized water and ethanol mixture (1 to 1 volume ratio). In batch, the average particle size of BSA@ZIF-8 is 1.1 µm (**Figure S12**).

4.2. AAT@ZIF-8 synthesis in flow

Solution A: AAT (6.1 mg) and 2-methylimidazole (1.05 g, 12.8 mmol) were added to a 10 mL volumetric flask. The solution was made up using deionized water.

Solution B: $Zn(OAc)_2 \cdot 2H_2O$ (0.878 g, 4.0 mmol) was added to a 50 mL volumetric flask. The solution was made up using deionized water.

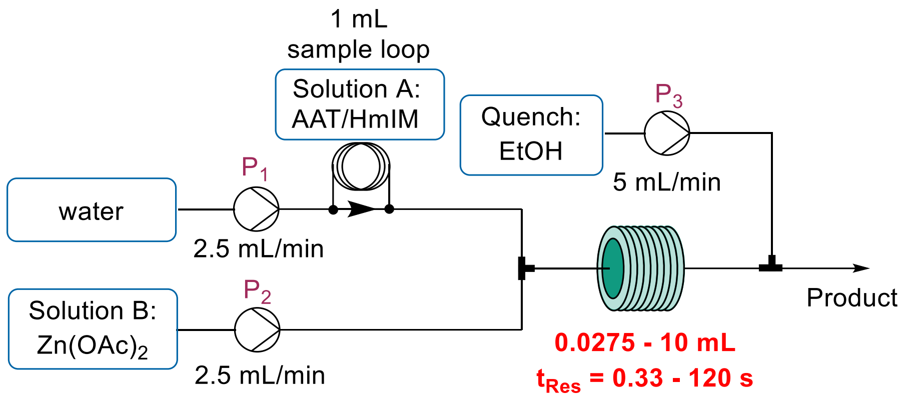

Figure S10. Schematic view of the flow setup for the synthesis of AAT@ZIF-8 samples, using pre-quench residence times of 0.33s, 30 s, or 120 s.

Procedure:

Water and Solution B were pumped directly through the reciprocating syringe pump at a flow rate of 2.5 mL/min each. Ethanol was pumped through the peristaltic pump at a flow rate of 5 mL/min. Solution A was loaded into a 1 mL sample loop, then injected. At the outlet, 3 × 2 mL samples were collected after the desired residence time, and the central one analyzed.

Between each sample collection, the tubing was cleaned by injecting a portion of acetic acid, followed by 2 portions of water through the sample loop. Once collected, the samples were stored at 4°C.

For comparison, the synthesis in batch were performed by mixing 1 mL of solution A and 1 mL of solution B. After 24h, the powder was recovered via centrifugation (5 min, 21000 rcf), washed (dispersion and centrifugation cycles) 3 times with deionized water and ethanol mixture (1 to 1 volume ratio), 1 time with 10% SDS aqueous solution, 3 times with deionized water and ethanol mixture (1 to 1 volume ratio) and stored at 4°C. In batch, the particle size of AAT@ZIF-8 ranges from 600 nm to 1 µm (**Figure S28**).

5. Quenching system optimization experiments

During the development of the flow quenching system, other substances were examined for their effectiveness in controlling the size of BSA@ZIF-8 particles.

5.1. BSA@ZIF-8 synthesis in flow without quench

BSA@ZIF-8 was synthesized in flow, to validate that the same crystalline form and size would be observed. As expected, the particles produced by both methods were comparable by SEM analysis (**Figure S12**).

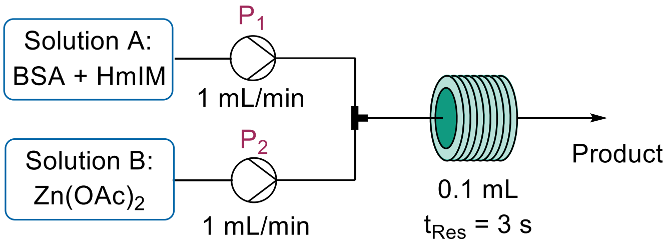

Figure S11. Schematic view of the flow setup for the synthesis of BSA@ZIF-8, with no quench.

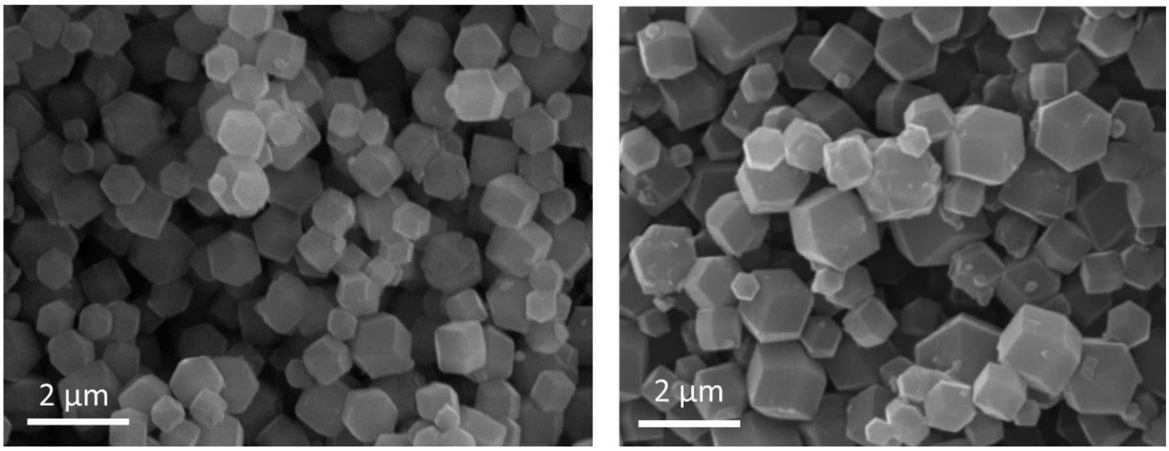

Figure S12. SEM images of BSA@ZIF-8. **Left:** sample produced in flow, using simple setup, giving average particle size of 0.9 µm; **Right:** sample synthesized in batch, giving an average particle size of 1.1 µm.

5.2. Dilution with water

To determine whether size control could be achieved by simply diluting the BSA@ZIF-8 mixture, samples were collected into a vial containing water, after a short residence time (0.33 s). No reduction in particle size was observed by SEM analysis (**Figure S14**).

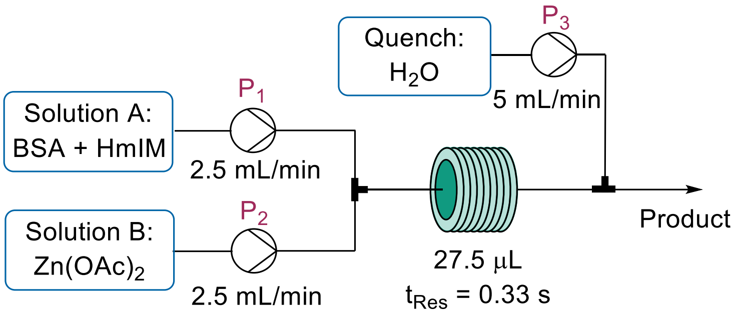

Figure 13. Schematic view of the flow setup for the synthesis of BSA@ZIF-8 with water dilution after 0.33 s residence time.

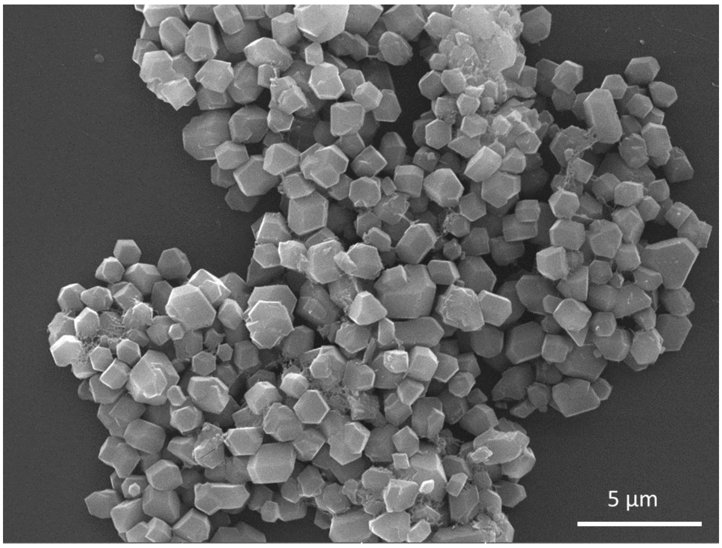

Figure S14. SEM image showing the size of particles formed, when the BSA@ZIF-8 mixture was mixed with water, after 0.33 s (27.5 µL residence time coil). Average particle size 1.2 µm.

5.3. Quench with 1-methylimidazole

It was envisioned that 1-methylimidazole could be used to modulate particle growth, as previously demonstrated in methanol.[10,11] Accordingly, samples were collected into an aqueous solution of 1-methylimidazole, after a short residence time (0.33 s).

The resulting particles (by SEM analysis, **Figure S16**) have an avergae particle size of 1 µm when using 1 equivalent (0.64 M 1-methylimidazole solution) and a slightly smaller average size (0.8 µm), when using 2 equivalents (1.28 M 1-methylimidazole solution). Therefore, the effect is expected to be insignificant unless an extremely large excess of 1-methylimidazole is present.

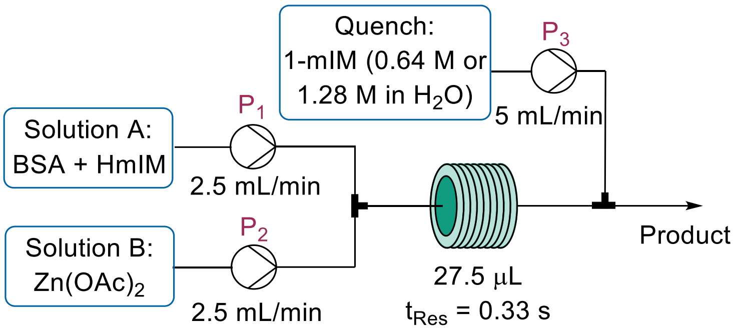

Figure S15. Schematic view of the flow setup for the synthesis of BSA@ZIF-8 with 1-methylimidazole quench after 0.33 s residence time.

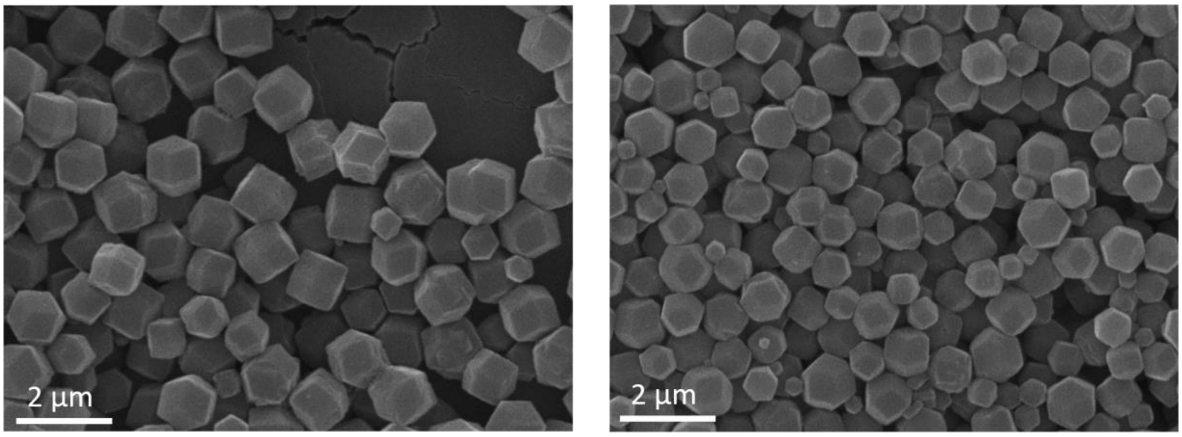

Figure S16. SEM images showing the size of particles formed, when the BSA@ZIF-8 mixture was mixed with an aqueous solution of 1-methylimidazole, after 0.33 s (27.5 µL residence time coil). **Left:** Quench using 0.64 M 1-methylimidazole solution (1 equivalent with respect to 2-methylimidazole), average particle size of 1 µm; **Right:** Quench using 1.28 M 1-methylimidazole solution (2 equivalents with respect to 2-methylimidazole), average particle size of 0.8 µm.

5.4. Quench with ethanol

To qualitatively determine the effect of quenching BSA@ZIF-8 mixture with ethanol, samples were collected into a vial containing ethanol, after a short residence time (0.33 s).

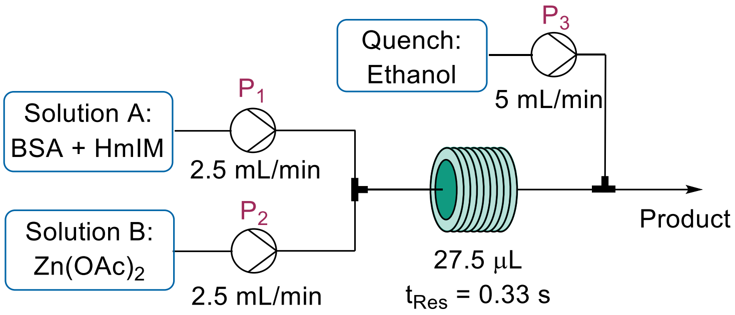

Figure S17. Schematic view of the flow setup for the synthesis of BSA@ZIF-8 with ethanol quench after 0.33 s residence time.

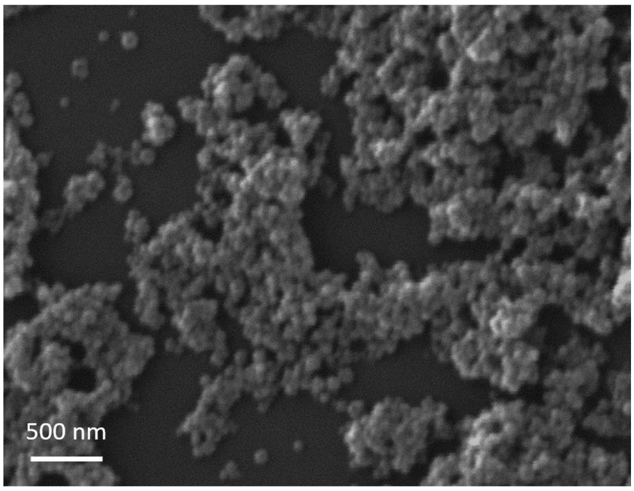

Figure S18. SEM images showing the size of particles formed, when the BSA@ZIF-8 mixture was mixed with ethanol after 0.33 s (27.5 µL residence time coil).

5.5. Optimization of ethanol flow rate

As described in the manuscript (**Figure 2b**), the flow rate of ethanol was varied, using a pre-quench residence time of 0.33 s.

Table S2. Tabulated results of study to determine the optimal flow rate of ethanol.

Entry	EtOH flow rate (mL/min)	Ratio EtOH:H_2O	Crystallite size (nm)[a]
1	0	0	>200
2	0.5	0.1	148.6
3	1	0.2	93.0
4	2.5	0.5	60.1
5	5	1	55.4
6	8	1.6	47.2

[a]Crystallite size determined by XRD.

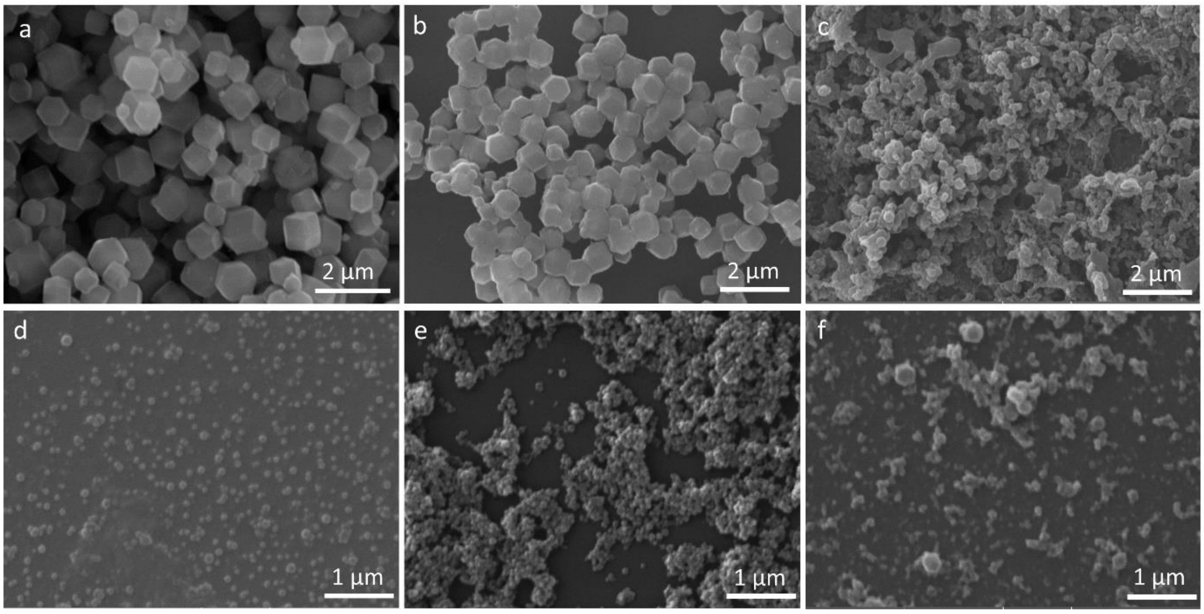

Figure S19. SEM micrographs of the BSA@ZIF-8 samples reported in **Table S2**. The samples were synthesized using ethanol flow rates of 0 mL/min, entry 1 (a); 0.5 mL/min, entry 2 (b); 1 mL/min, entry 3 (c); 2.5 mL/min, entry 4 (d); 5 mL/min, entry 5 (e); 8 mL/min, entry 6 (f).

6. Analysis of BSA@ZIF-8 produced using varied residence times

The particle suspensions were drop cast on clean (100) Si. AFM topography in tapping mode was measured in 3 different regions of each sample. The particle size was measured considering the vertical height of the supported particles, and performing a statistical analysis (N>90) to obtain the particle height distribution. Larger agglomerates composed of many particles were selectively excluded from the analysis to obtain a realistic value of the particle size. Histograms were fit with a Gaussian distribution and the average values were obtained from the fit parameters (see example in **Figure S20**). Representative samples including topography, amplitude and phase images are shown in **Figure S21**.

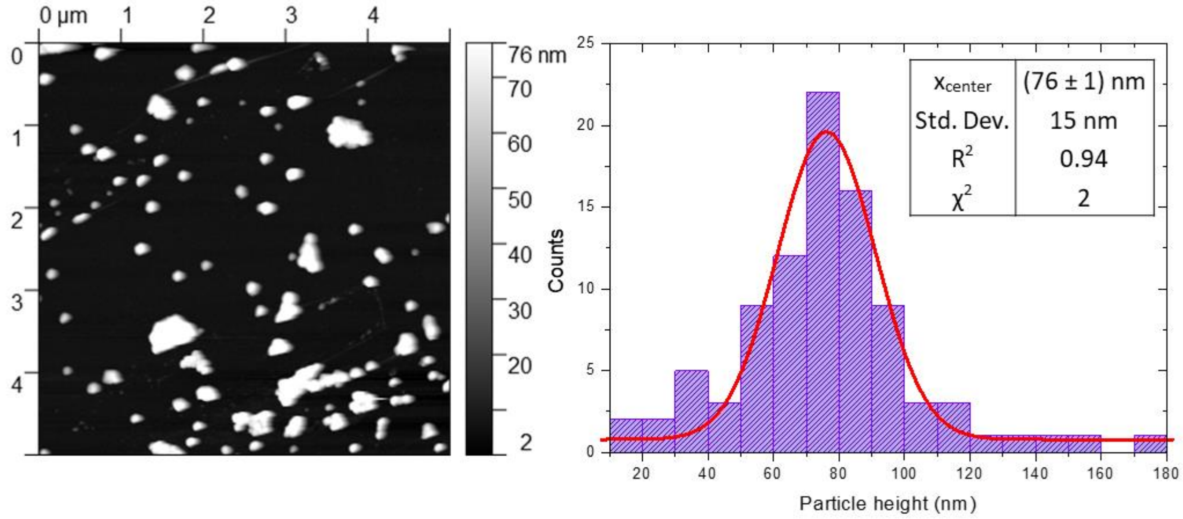

Figure S20. Left: 5 × 5 µm^2 AFM topography measured on a sample synthesized with t_{Res}=30 s. **Right:** Corresponding histogram of particle sizes obtained from the data extracted from the same topography, including Gaussian fit of the data. The resulting fit parameters for this particular sample are shown on the inset of the figure.

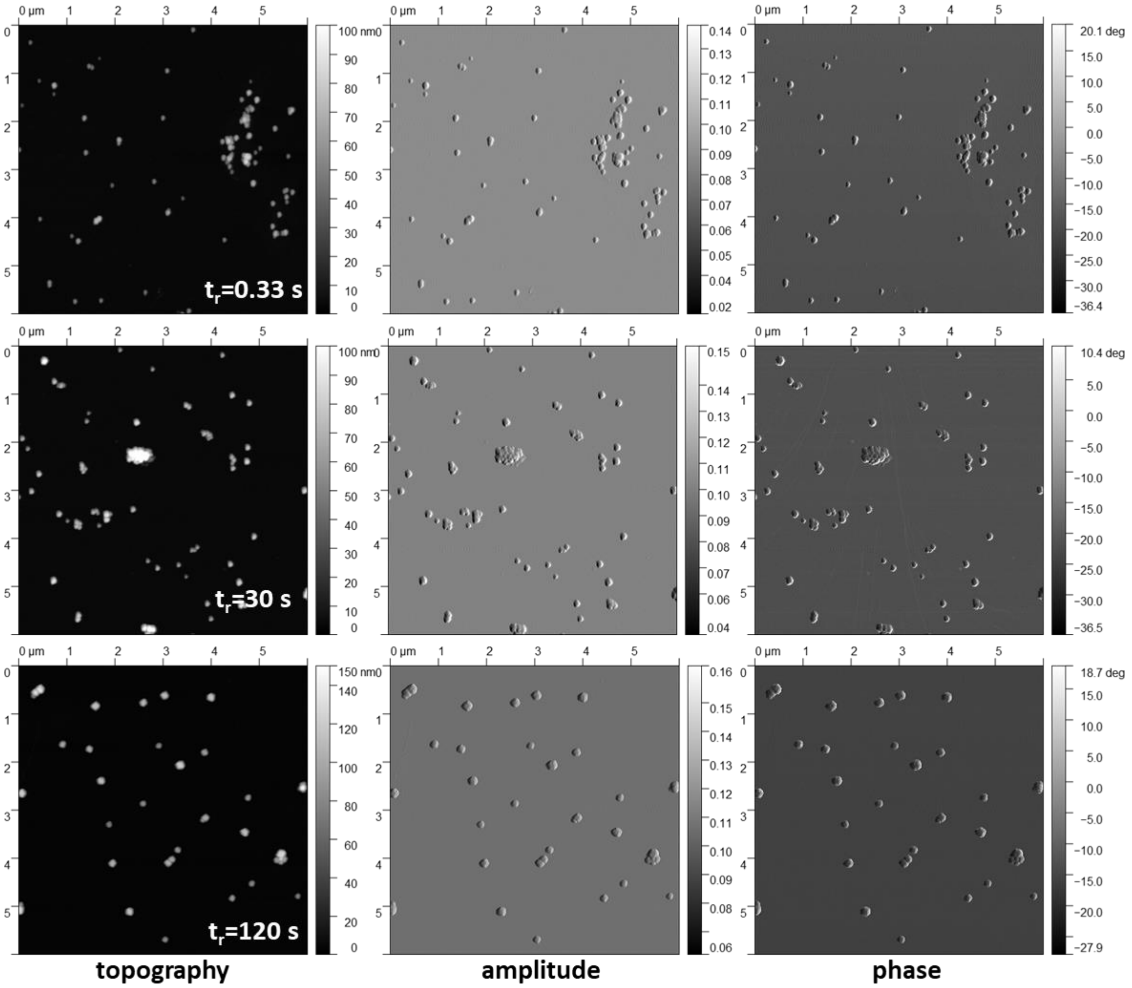

Figure S21. AFM topography, amplitude and phase images from representative samples corresponding to residence times of 0.33 s, 30 s and 120 s. The agglomerated particles seen on the first and second examples were excluded from the analysis.

The analysis included 9 repeated samples synthesized using the same protocol and pre-quench residence time (t_{Res}) for 0.33 s, 12 s and 60 s (3 for each t_{Res}) and 4 repeated samples for 30 s and 120 s (2 for each t_{Res}), showing a good correlation between all sets of repeated samples (**Figure S22a**). There is an overall trend to increase the particle size with t_{Res}, which can be qualitatively modeled with a power law of the form $\mathrm{h} \propto \mathrm{t}_{\mathrm{Res}}^{0.6}$ (h: average particle size). This could serve as a “calibration curve” for the method. The relative standard deviation, calculated as σ/h, was obtained for each sample from the statistical fits (**Figure S22b**) and represents the degree of polydispersity of the particle size distributions. The obtained values appear to remain approximately constant at 30% of the average value.

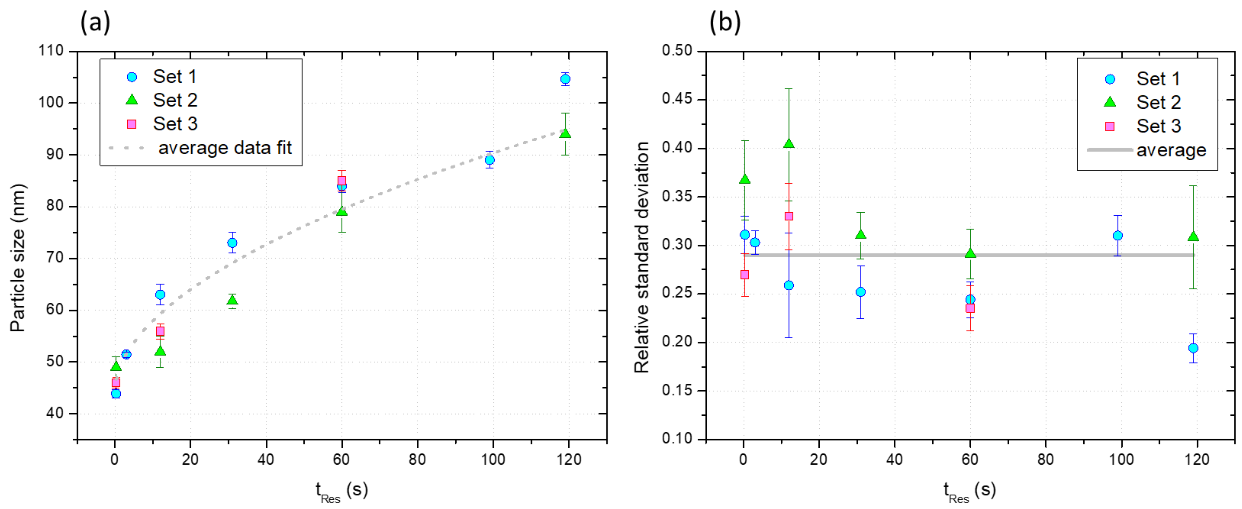

Figure S22. (a) Particle size (h) vs. residence time obtained from three different sets of repeated samples. The dotted gray line represents the fit obtained for the average data. (b) Relative standard deviation, calculated as σ/h, as a function of the residence time, including a plot of the average value of the data (gray line).

The obtained values from all repeated samples corresponding to the same residence time were averaged to obtain a representative plot, which was subsequently fit with a power law, as shown on **Figure 2c** of the manuscript. The corresponding data is shown in **Table S3**.

Table S3: Average particle size (obtained from AFM analysis) and average crystallite size (obtained from XRD analysis) vs. residence time.

Residence time (s)	Particle size (nm)	Particle size standard error (nm)	Crystallite size (nm)
0.33	46	2	37
3	52	1	-
12	57	3	-
30	67	6	59
60	83	4	-
99	89	2	-
120	99	5	95

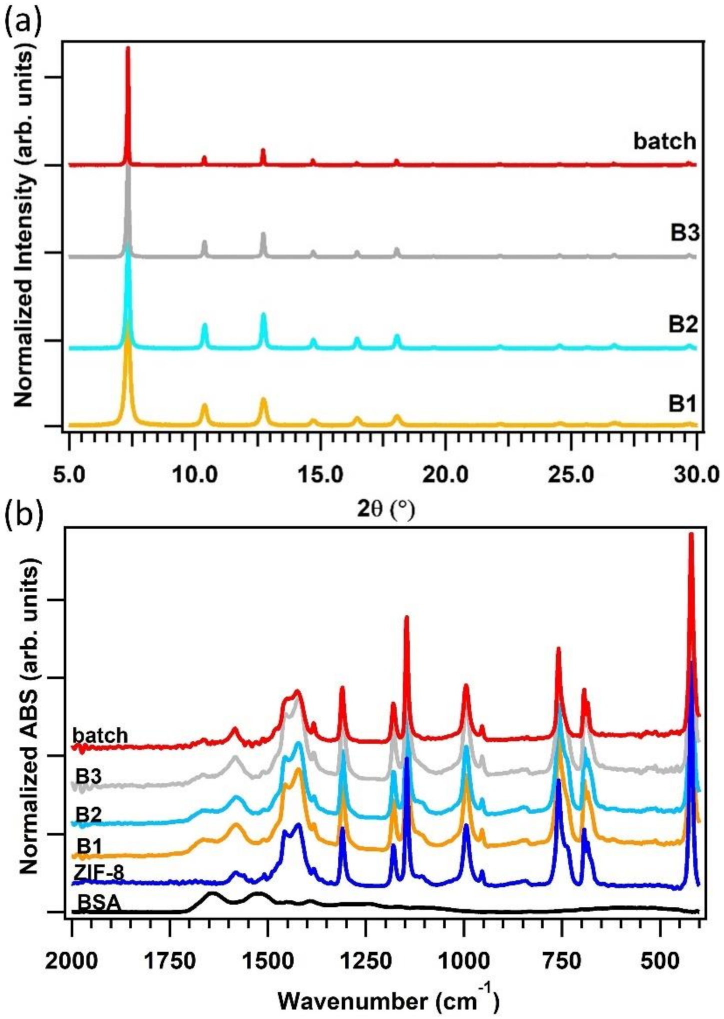

Figure S23: . XRD patterns (a) of BSA@ZIF-8 synthesized in batch and in flow (B1, B2, B3). FTIR spectra (b) of BSA@ZIF-8 synthesized in batch and in flow (B1, B2, B3) and of ZIF-8 and BSA.

Table S4: BSA weight % (wt%) calculated from ICP quantification of sulfur in the dry powders.

Residence time (s)	BSA wt%
0.33	5.1 ± 0.7
30	5.2 ± 0.6
120	5.8 ± 0.4
Batch	4.5 ± 0.4

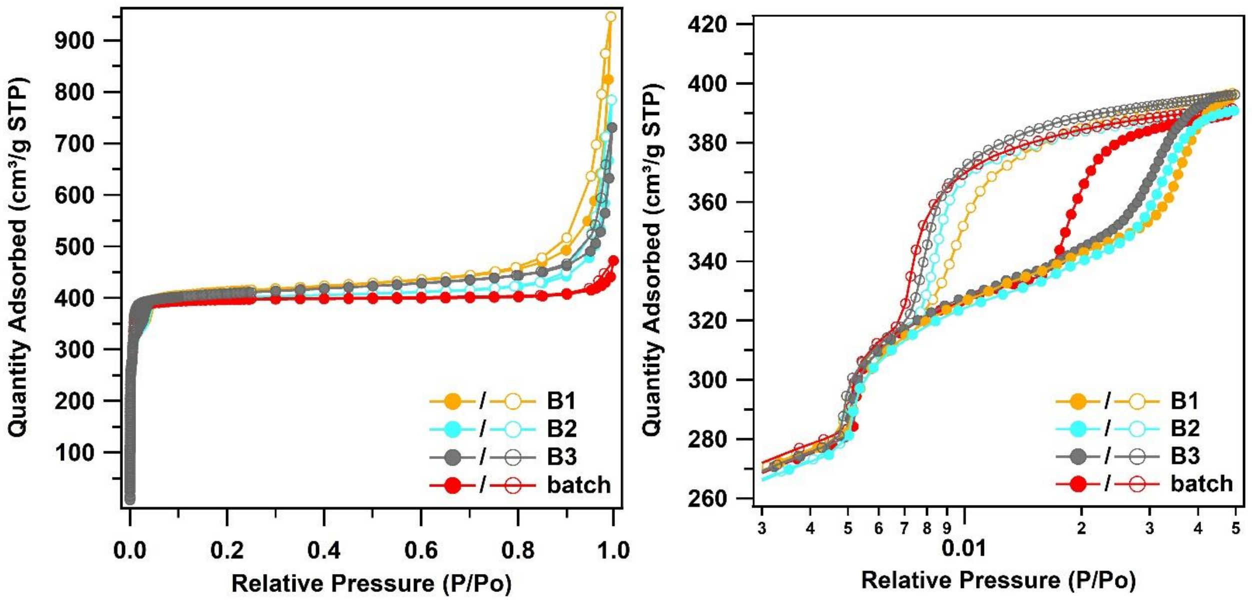

Figure S24: N_2 77K sorption isotherms of the BSA@ZIF-8 synthesized in batch and in flow (B1, B2, B3). The calculated BET surface area are: 1498±10 m^2/g (C=1905, 0.9998) for BSA@ZIF-8 synthesized in batch; 1539±2 m^2/g (C=931, Correlation Coefficient=0.999995) for B1; 1518±5 m^2/g (C=1036, Correlation Coefficient=0.99996) for B2; 1524±2 m^2/g (C=1103, Correlation Coefficient=0.99993) for B3. Gate adsorption behavior observed at a relative pressure of around 10^{-2} is highlighted in the right plot. The dependence of the gate opening pressure on the particle size is similar to the trends previously reported in the literature[12,13] and the values are reported in the **Table S5**.

Table S5: Gate opening and closing relative pressure values of the BSA@ZIF-8 synthesized in batch and in flow (B1, B2, B3).

**Sample**	**Gate opening relative pressure**	**Gate closing relative pressure**
B1	0.035	0.010
B2	0.033	0.009
B3	0.030	0.008
batch	0.020	0.007

6.1. Measure of BSA@ZIF-8 stability in stock solution

The stability of the particles stored in the stock solution at room temperature (directly from synthesis) was studied for the smallest particles. The particle size from a drop cast sample corresponding to t_{Res}=0.33 s was compared to a sample drop cast from the same stock solution 2 weeks later. The results confirmed that the particle size was conserved with no apparent degradation or agglomeration over that period of time (**Figure S25**).

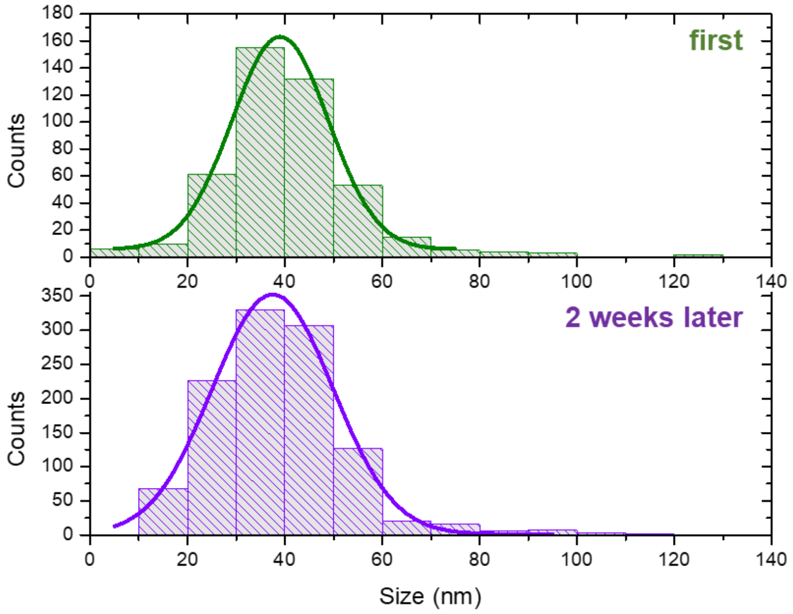

Figure S25. Analysis of stability over time for a sample synthesized with residence time 0.33 s. The histogram on the top corresponds to the fresh sample, while the histogram on the bottom corresponds to a sample drop cast from the same stock solution 2 weeks later, showing that the average particle size is preserved after this time.

7. Analysis of AAT@ZIF-8 produced using varied residence times

The particle size was examined by AFM for the AAT@ZIF-8 particles synthesized with the selected residence times, 0.33 s, 30 s and 120 s (referred to in the manuscript as A1, A2 and A3 respectively). The AFM results obtained from these samples are shown in **Figure S26**.

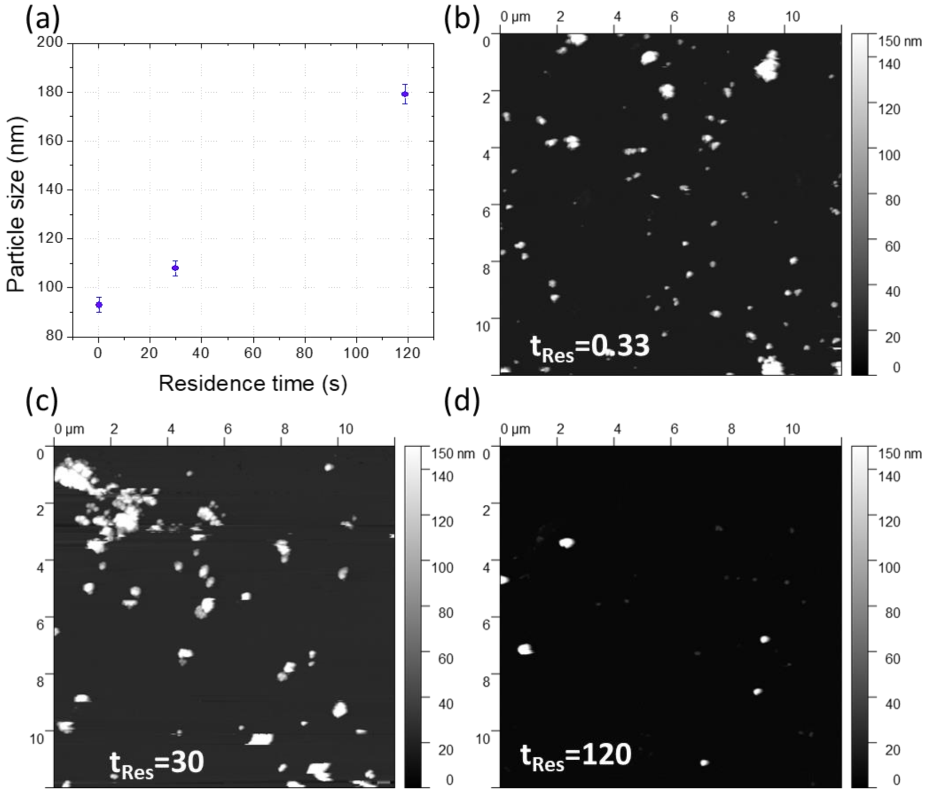

Figure S26. AAT@ZIF-8 particle sizes obtained from AFM measurements. (a) Average particle size obtained for samples A1, A2 and A3 as a function of residence time. (b), (c) and (d) representative AFM topographies from each sample corresponding to residence times 0.33 s, 30 s and 120 s respectively. Agglomerates such as the ones observed in (b) and (c) were excluded from the analysis.

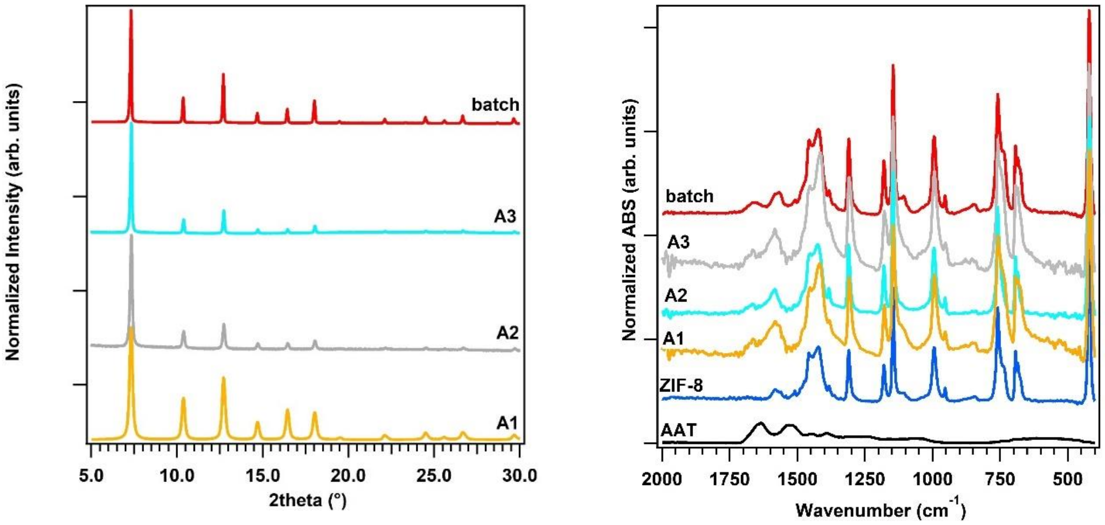

Figure S27. Left: XRD patterns of AAT@ZIF-8 samples synthesized in batch and in flow (A1, A2 and A3). **Right:** FTIR spectra of AAT@ZIF-8 synthesized in batch and in flow (A1, A2, A3) and of ZIF-8 and AAT.

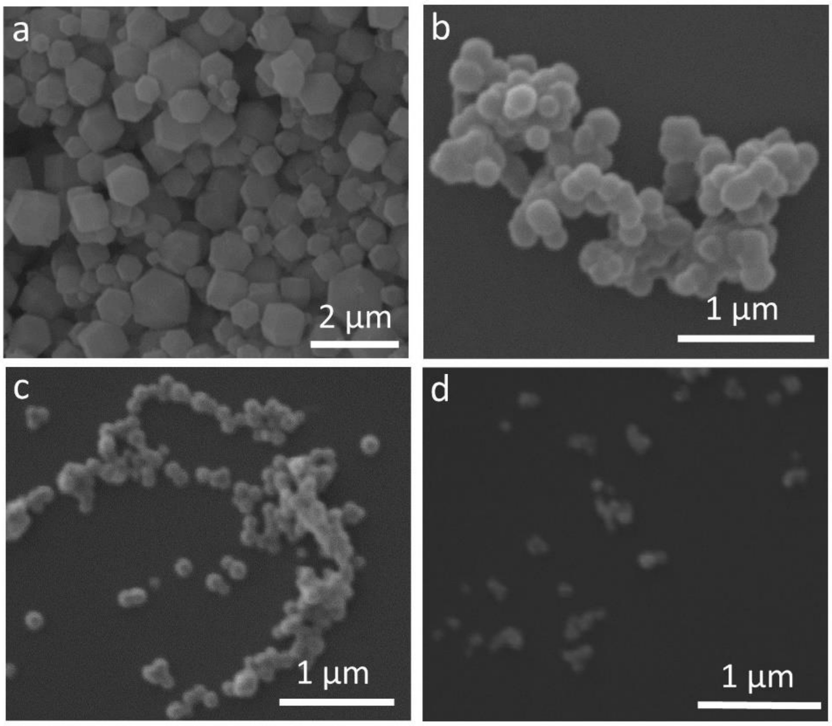

Figure S28. SEM micrographs of AAT@ZIF-8 particles obtained in batch (a) and synthesized in flow with different pre-quench residence times: A3, t_{Res}=120 s (b), A2, t_{Res}= 30 s (c), A1, t_{Res}= 0.33 s (d).

Table S6: AAT weight % (wt%) calculated from ICP quantification of sulfur in the dry powders.

Residence time (s)	AAT wt%
0.33	4.2 ± 0.3
30	4.1 ± 0.7
120	3.1 ± 0.6
Batch	3.1 ± 0.1

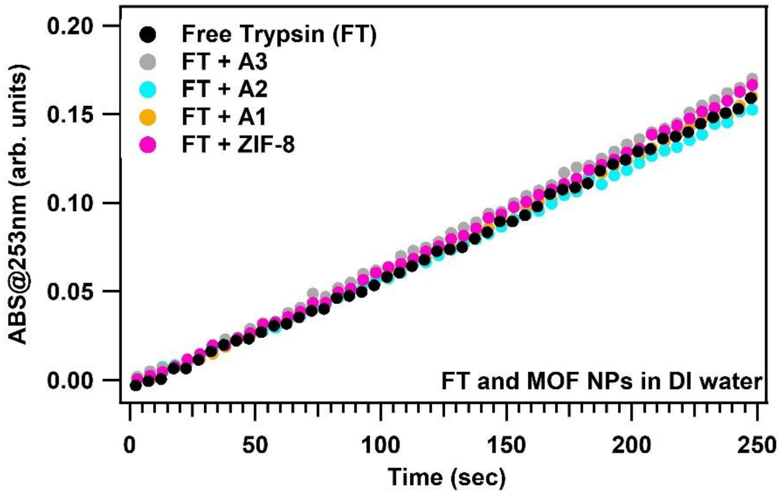

Figure S29: Trypsin protease activity results of trypsin and of trypsin exposed to AAT@ZIF-8 samples (flow syntheses) and to a pure ZIF-8 sample (batch synthesis) in DI water for 30 minutes at 4°C.

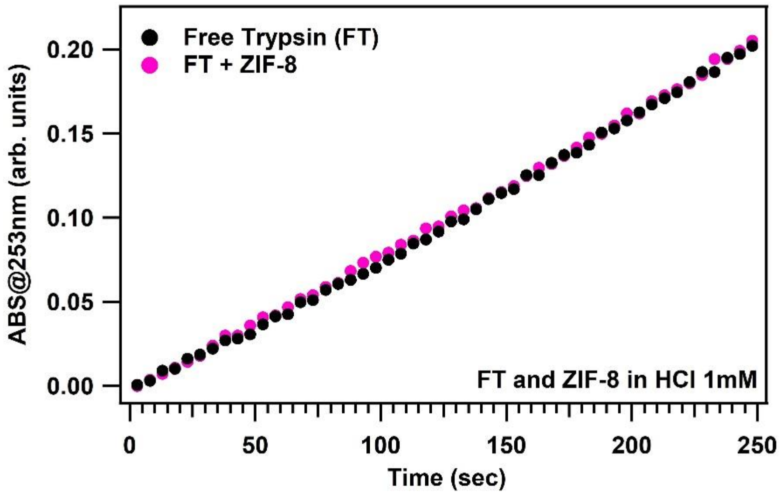

Figure S30: Trypsin protease activity results of trypsin and of trypsin exposed to a solution of degraded pure ZIF-8 sample (batch synthesis, degraded for 1 hour in HCl 1mM) in HCl 1mM for 30 minutes at 4°C. The activity of trypsin is comparable in the two cases, demonstrating that it is not influenced by the presence of the decomposition products of the pure MOF.

8. Scalability of the synthetic method

In order to demonstrate the scalability of this method, one particle size of BSA@ZIF-8 were targeted (t_{Res} = 30 s corresponding to sample B2).

These experiments were performed as follows, using larger reagent stock solutions and a wider tubing diameter (1.6 mm), placed in a sonic bath. The peristaltic pump (ethanol feed) was replaced by an HPLC pump (Knauer Azura Compact, up to 10 mL/min) for this longer demonstration.

Solution A: BSA (495 mg) and 2-methylimidazole (78.75 g, 959 mmol) were added to a 750 mL volumetric flask. The solution was made up using deionized water.

Solution B: $Zn(OAc)_2 \cdot 2H_2O$ (13.17 g, 60 mmol) was added to a 750 mL volumetric flask. The solution was made up using deionized water.

Solutions A and B were pumped directly through the reciprocating syringe pump at a flow rate of 2.5 mL/min each. Ethanol was pumped through the HPLC pump at a flow rate of 5 mL/min.

The reactor was allowed to equilibrate for 3 residence times, then material was collected continuously for 5 hours, into a new flask every 30 minutes. A separate sample of 10 mL (1 minute) was also collected for analysis every 30 minutes. A total of 3 L output solution was collected.

A representative portion of 32 mL was taken from 6 of the separate collection flasks (192 mL total, 6.4% of the total processed volume) and the solids were separated by centrifugation, then washed with ethanol (3 × 5 mL) and dried overnight in a vacuum oven at 40 °C.

The combined mass of BSA@ZIF-8 recovered from these fractions was 666 mg, corresponding to 10.4 g when extrapolated to the total processed volume. This represents a productivity of 2.08 g/h.

Over time, the average crystallite size (calculated from XRD patterns of samples collected every 30 minutes) was stable (see **Table S7**).

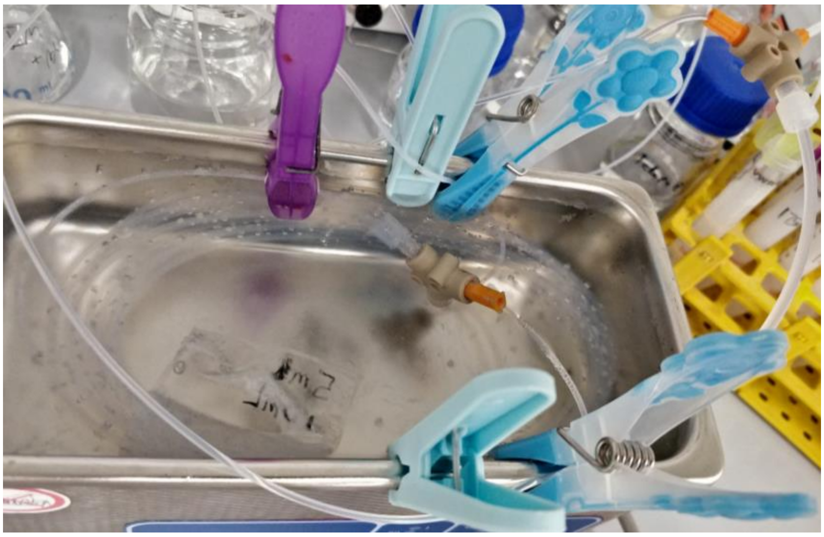

Figure S31. Photograph of residence time coil (t_{Res}=30 s) in a sonic bath, used to produce material during prolonged processing time (5 h), as a measure of scalability of the method.

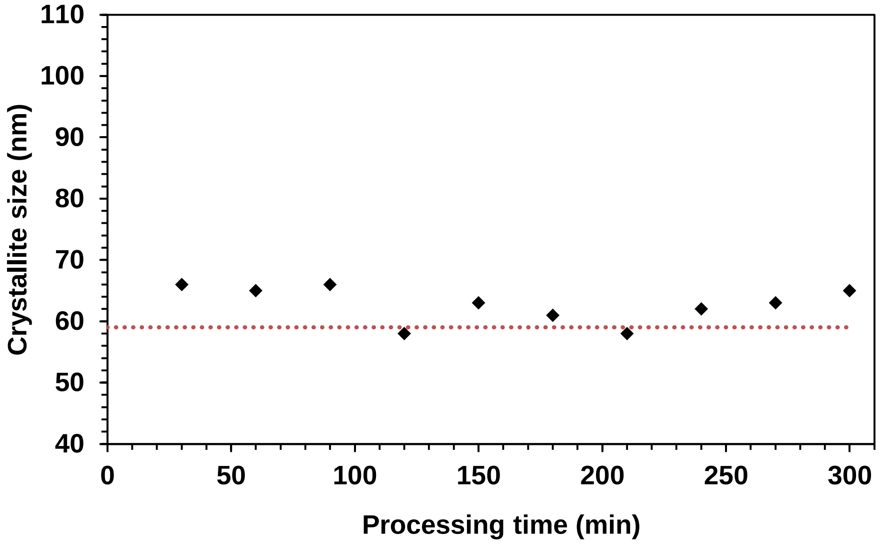

Figure S32. Graph of crystallite size over time, during the 5 hour long run, determined by XRD analysis of the samples taken every 30 min. The orange line represents the crystallite size for this residence time found by XRD analysis in small scale synthetic tests (59 nm, see Table S2). The values from this graph are tabulated below (**Table S7**).

Table S7. Analysis of the average crystallite size (by XRD, see Figure S26) in samples collected during a 5 hour continuous synthesis of BSA@ZIF-8, using 30 seconds as residence time.

Time on stream (min)	Crystallite size (nm)
30	66
60	65
90	66
120	58
150	63
180	61
210	58
240	62
270	63
300	65

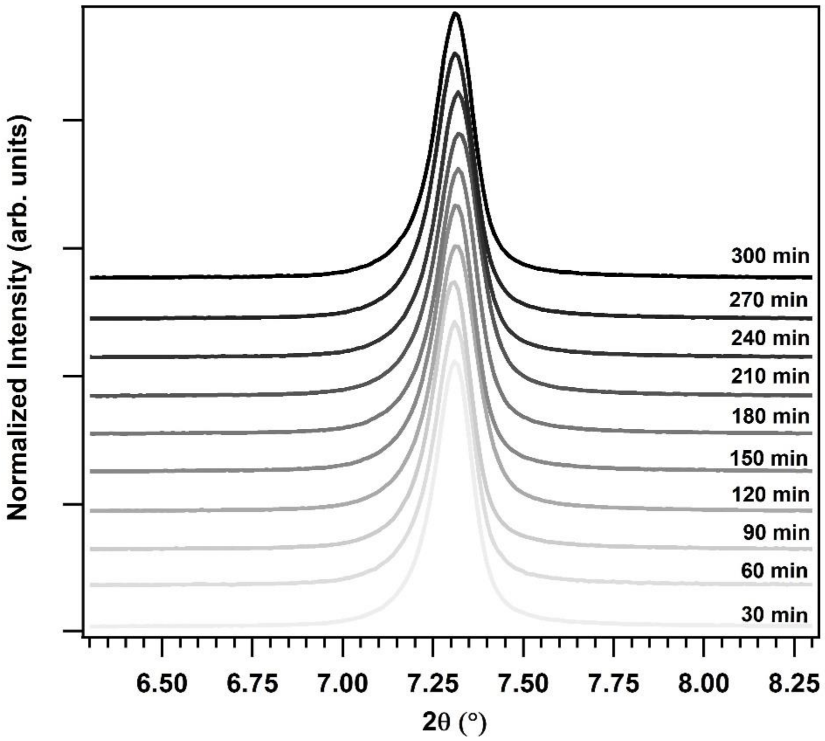

Figure S33. XRD patterns (reported in the 6.3° to 8.3° region to highlight the (110) peak of ***sod*** ZIF-8) of the BSA@ZIF-8 samples reported in **Table S5** and synthesized using a residence time of 30 s and collected every 30 minutes (total collection time 5 h) on stream.

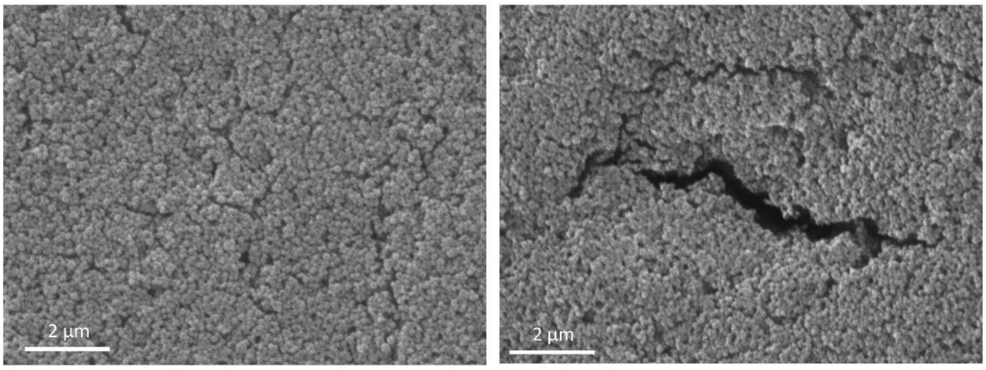

Figure S34: SEM micrographs of the BSA@ZIF-8 samples collected after 30 minute (left) and 5 hours (right) on stream.

9. References

[1] H. Amenitsch, M. Rappolt, M. Kriechbaum, H. Mio, P. Laggner, S. Bernstorff, *J Synchrotron Rad* **1998**, *5*, 506–508.
[2] I. Grillo, *Current Opinion in Colloid & Interface Science* **2009**, *14*, 402–408.
[3] S. Cao, T. D. Bennett, D. A. Keen, A. L. Goodwin, A. K. Cheetham, *Chem. Commun.* **2012**, *48*, 7805–7807.
[4] "Procedure for Enzymatic Assay of Trypsin (EC 3.4.21.4)," can be found under https://www.sigmaaldrich.com/technical-documents/protocols/biology/enzymatic-assay-of-trypsin.html
[5] K. S. Park, Z. Ni, A. P. Côté, J. Y. Choi, R. Huang, F. J. Uribe-Romo, H. K. Chae, M. O'Keeffe, O. M. Yaghi, *PNAS* **2006**, *103*, 10186–10191.
[6] J.-M. Commenge, L. Falk, *Chemical Engineering and Processing: Process Intensification* **2011**, *50*, 979–990.
[7] J. M. Reckamp, A. Bindels, S. Duffield, Y. C. Liu, E. Bradford, E. Ricci, F. Susanne, A. Rutter, *Org. Process Res. Dev.* **2017**, *21*, 816–820.
[8] X. Wu, H. Yue, Y. Zhang, X. Gao, X. Li, L. Wang, Y. Cao, M. Hou, H. An, L. Zhang, et al., *Nat Commun* **2019**, *10*, 1–8.
[9] W. Liang, R. Ricco, N. K. Maddigan, R. P. Dickinson, H. Xu, Q. Li, C. J. Sumby, S. G. Bell, P. Falcaro, C. J. Doonan, *Chemistry of Materials* **2018**, *30*, 1069–1077.
[10] J. Cravillon, R. Nayuk, S. Springer, A. Feldhoff, K. Huber, M. Wiebcke, *Chemistry of Materials* **2011**, *23*, 2130–2141.
[11] T. Enomoto, S. Ueno, E. Hosono, M. Hagiwara, S. Fujihara, *CrystEngComm* **2017**, *19*, 2844–2851.
[12] S. Watanabe, S. Ohsaki, T. Hanafusa, K. Takada, H. Tanaka, K. Mae, M. T. Miyahara, *Chemical Engineering Journal* **2017**, *313*, 724–733.
[13] C. Zhang, J. A. Gee, D. S. Sholl, R. P. Lively, *J. Phys. Chem. C* **2014**, *118*, 20727–20733.
